# Sustainable livelihood capital and climate change adaptation in Pakistan's agriculture: Structural equation modeling analysis in the VIABLE framework

**DOI:** 10.1016/j.heliyon.2023.e20818

**Published:** 2023-10-13

**Authors:** Muhammad Mobeen, Khondokar H. Kabir, Uwe A. Schneider, Tauqeer Ahmed, Jürgen Scheffran

**Affiliations:** a Research Group Climate Change and Security (CLISEC), Institute of Geography, University of Hamburg, Germany; b Research Unit Sustainability and Climate Risks, Center for Earth System Research and Sustainability (CEN), University of Hamburg, Germany; c School of Environmental Design and Rural Development, University of Guelph, Canada; d Department of Agricultural Extension Education, Bangladesh Agricultural University, Mymensingh, Bangladesh; e School of Integrated Climate System Sciences (SICSS), University of Hamburg, Germany; f Department of Sociology and Criminology, University of Sargodha, Sargodha, Pakistan; g Department of Earth Sciences, University of Sargodha, Sargodha, Pakistan

**Keywords:** Capital, Adaptation, VIABLE framework, Agriculture, Pakistan

## Abstract

This study aims to assess the role of sustainable livelihood capital, the mediation of investments and farming purposes, and the moderation of climatic and non-climatic factors in the adaptation process, particularly in the aspects of Crop, Farm, Irrigation, and Economic Management. Moreover, guided by the VIABLE (Values and Investments for Agent-Based Interaction and Learning in Environmental Systems) framework, we analyze stakeholders' actions, priorities, and goals in the climate change adaptation process. A structured questionnaire was designed based on a five-point Likert scale covering the concepts of livelihood capital, climate change adaptation, investment priorities, farming constraints, and farmers' decision-making factors. Field data were collected from 800 farmers during December 2021 to February 2022 in the irrigated agricultural regions in the Indus Plain of the Punjab and Sindh provinces, Pakistan. We employed the Partial Least Square Structural Equation Modeling approach to the VIABLE framework (VIABLE-SEM) to analyze the collected data. The results confirm livelihood capital as the most significant determinant (beta = 0.57, effect size = 0.503) for farmers’ adaptation strategies in the Indus plain. Other variables, such as the principal purpose of farming, available investment options, natural and human constraints, appear less important. We identified 13 significant viability pathways that show investment priorities, farming purposes, and constraints faced by the farmers in climate change adaptation. The study also found that non-climatic factors negatively influence (beta = −0.156) the relationship between capital and adaptation, while climatic factors positively influence (beta = 0.050) this relationship. Interestingly, the presence of these influencing factors increases the adaptive capacity of farmers. These findings have important implications for policymakers and researchers in designing and implementing effective climate change adaptation strategies in the agricultural sector of Pakistan.

## Highlights

• The VIABLE-SEM model explains 62.2% of farmers' adaptation strategies in Pakistan's irrigated agriculture.
• Livelihood capital alone accounts for 57 % of the adaptation process; ther factors play a lesser role.
• Non-climatic factors impede the capital-adaptation relationship, whereas climatic factors bolster it.
• The presence of influencing factors increases the adaptive capacity of farmers.

## Graphical abstract

**Figure:**
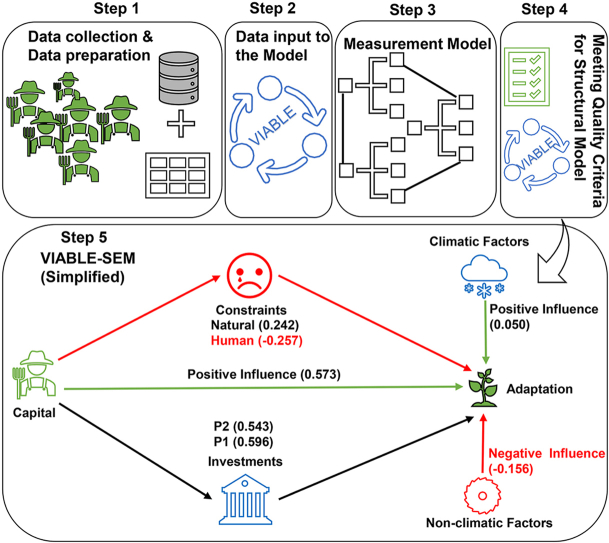

## 1 Introduction

### 1.1 Climate change adaptation in Pakistan's agriculture

According to the global climate risk index, Pakistan was ranked as the fifth most climate-affected country from 1999 to 2018 [1]. Climate risk is estimated to increase further if the temperature rises to 2–3° by 2050 [2]. The country's agricultural sector is more vulnerable to climate risk due to its reliance on water and temperature [3]. Studies reported that Pakistan already suffers from noticeable impacts of climate change, including floods, droughts, heat waves, and erratic rainfall [4,5]. The domestic food supply is already under stress due to reduced crop yields caused by climate change [6]. Farmers respond to climate change in multiple ways [7]. Adaptation of agricultural practices can reduce losses in rural livelihoods and agricultural productivity thus alleviating adverse effects of climate change [8,9] on individual farms and agricultural communities [10,11]. While the adaptation of farming practices to climate change is a widespread response in the agriculture sector, not all individuals do it effectively, resulting in unfavorable outcomes [12,13]. The existing body of research requires further exploration into understanding the role of Sustainable Livelihood Capital for climate change adaptation in this critical sector. This is particularly true within the unique context of the irrigated regions of the Indus plain. The aim of this study is to address this deficit.

Many recent studies on climate change adaptation have focused on the agricultural sector [[14], [15], [16], [17]]. Some presented climate change assessments on agricultural practice and its productivity [[18], [19], [20]] and some on mitigation studies [21]. This article investigates the role of sustainable livelihood capital, the mediation of investments and farming purposes, and the moderation of climatic and non-climatic factors in the adaptation process by developing the VIABLE framework (Values and Investments for Agent-Based Interaction and Learning in Environmental Systems) which has three main contributions. First, from a brand-new perspective, the findings from this study based on the VIABLE framework reveal that livelihood capital as the most significant determinant for farmers' adaptation strategies in the Indus plain. Other variables, such as the principal purpose of farming, available investment options, natural and human constraints, appear less important. This study identified 13 significant viability pathways that show investment priorities, farming purposes, and constraints faced by the farmers in climate change adaptation. The study also found that non-climatic factors negatively influence the relationship between capital and adaptation, while climatic factors positively influence this relationship. Interestingly, the presence of these influencing factors increases the adaptive capacity of farmers. These findings offer empirical evidence for the VIABLE framework which is a supplement for this research domain, and have significant implications for policymakers and researchers in designing and implementing effective climate change adaptation strategies in the agricultural sector of Pakistan. Second, studies with farm surveys are limited to comparatively small areas and sample sizes. To overcome the limitations of small samples, we surveyed a relatively large area of irrigated regions in Pakistan and collected empirical data (N = 800) on farmer's adaptation decisions, their capital, priorities for investing in crop, land, and water, and goals of farming such as profit maximization, subsistence, social status, and competition with neighboring farmers; constraints farmers face; and factors influencing their decision. Third, many studies assess the adaptation process as a linear causal relationship dependent upon one or two variables that ignore the influence of other intervening variables, such as investment priorities, farming goals, and constraints. To address the limits of a linear depiction of adaptation processes, we develop a comprehensive structural equation model based on the VIABLE (Values and Investments from Agent-Based interaction and Learning in Environmental systems) model framework with the role of livelihood capital as a predictor, investment priorities of farmers, farming goals, constraints as a mediator, and factors affecting farming decisions as moderator. Despite a large body of scientific literature on climate change adaptation, only a few studies incorporate other intervening variables like investment priorities, the purpose of farming, and constraints of farming in making adaptive decisions [22]. Comprehensive empirical farm-level estimations for understanding the role of these variables are scarce [21,23]. Little is known from previous literature when attempting to model the adaptation process in the presence of multiple variables under the climatic and non-climatic factors of farmers' decision-making. This research aims to address these gaps.

We used a sustainable livelihood framework [24] to explore how livelihood capital can lower climate change risks and vulnerabilities [25,26]. This framework identifies five key types of capital (human, social, natural, physical, and financial) that people need to maintain their sustainable livelihoods. We use capital as a cumulative measure that represents human, social, natural, and financial capital as one variable. Capital is the capability of farmers to enable them to make decisions. In adaptation, the capital provides the resources, opportunities, and necessary skills to adapt to the changing climatic conditions, which are strongly linked to adaptive capacity [27]. Different types of livelihood capital influence agricultural decision-making and the choice of livelihood strategy [9,28,29].

In our analysis, we apply the VIABLE framework, which combines actors’ capabilities, action priorities, values, and goals along with the feedback they receive in response to their actions and environmental changes [[30], [31], [32]].

This study attempts (1) to evaluate the role of Sustainable Livelihood Capital for agricultural adaptation to climate change in the Indus plain; (2) to highlight the pathways of farmers’ adaptation options investment priorities, their purpose of farming, and constraints they face in the adaptation process; (3) to evaluate the influence of climatic and non-climatic factors on adaptation actions.

## 2 Theoretical background

### 2.1 Hypothesis development

The concept of sustainable livelihood framework [24] was used to explore how livelihood capital can lower climate change risks and vulnerabilities [25,26]. This framework identifies five key types of capital (human, social, natural, physical, and financial) that people need to maintain sustainable livelihoods. Capital is the capability of farmers to enable them to take a decision. In adaptation, the capital provides the resources, opportunities, and necessary skills to adapt to the changing climatic conditions, which are strongly linked to adaptive capacity [27]. Different types of livelihood capital influence agricultural decision-making and the choice of livelihood strategy [9,28,29]. In this study, human, social, natural, and financial capital were combined to form a single variable representing the farmers' capabilities.

According to Pretty and Ward [33], livelihood capital, investment opportunities, farming goals, financial resources, and other constraints are critical factors for farming decisions. McDowell and Hess [34] reported that the endowment with livelihood capital limits adaptation options and increases vulnerability to climatic variability. As we stated, investment priorities, goals, constraints, and factors are equally important. Therefore, a better understanding of these variables can provide a comprehensive policy action to respond to climatic changes [11].

Therefore, we stated our null hypothesis H0 that “No significant relationship is found by taking farmers' capital as an independent variable, investment priorities, farming purpose and as a mediator, factors of farmers decisions as moderator and adaptation as an outcome”. For exploring the role of livelihood capital, we state H1 that “Significant relationships exist by taking farmers’ capital as an independent variable, investment priorities, farming purpose and constraints as a mediator and factors as moderator and adaptation as an outcome. We refer here study by Malek et al. [35] which found that farmers' capital investments in more efficient irrigation technologies can significantly improve the agricultural economy, especially in the context of climate change adaptation. Li et al. [36],Saptutyningsih and Dewi Nurcahyani [37] highlighted that social capital has the significant role in climate change adaptation actions. These studies provide robust foundation for our hypotheses H0 and H1 testing the role of different factors in climate change adaptation.

To understand the role of intermediate variables, we state our mediation hypothesis H2 as “the investment options and farming purpose and constraints mediate the relationship between capital and adaptation. The complexity of the adaptation process is underscored by several studies. As Lobell et al. [38] elucidated that investments intended to enhance climate change adaptation tend to be context-specific, favoring certain crops and regions over others. This suggests that the effectiveness of investment options is not uniform but varies depending on the specific context. Furthermore, Okada et al. [39] provided evidence that investments in water could lead to positive results in crop yield. However, the potential benefits were not without challenges. Specifically, Ozor et al. [40] highlighted land as a significant constraint preventing farmers to adapt. Abid, Schneider and Scheffran [8] identified finances and resources as key adaptation constraints. Collectively, these studies underscore the multifaceted nature of the adaptation process and provide a solid foundation for further investigation of our hypothesis H2. To explore how external factors are influencing the farmers' decisions, we stated our moderation hypothesis H3 as “Climatic factors and Non-climatic factors moderate the relationship between capital and adaptation.” This hypothesis is grounded in the work of Karki, Burton and Mackey [41] who conducted a study in Nepal and found that both climatic and non-climatic factors posed a significant direct threat to the livelihoods of rural farmers who are heavily reliant on natural resources. However, it is important to note that non-climatic factors also play an important role in shaping adaptation practices. To gain a more nuanced understanding of the moderating effect, we have subdivided H3 into H3a and H3b. Therefore, we state H3a as “Climatic factors moderate the relationship between capital and adaptation” and H3b as “Non-climatic factors moderate the relationship between capital and adaptation”.

### 2.2 The VIABLE framework

**Fig. 1:**
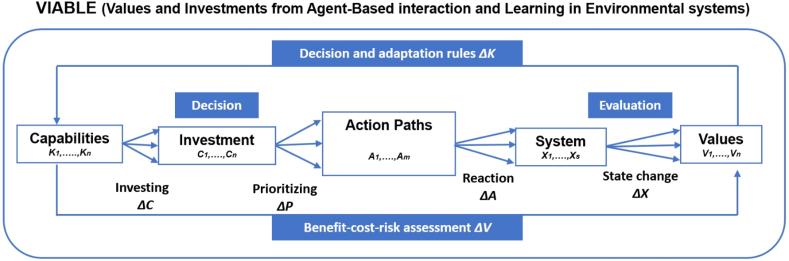
Schematic diagram of the VIABLE framework.

The VIABLE modeling approach is rooted in viability theory which looks into the development of constrained dynamic systems [42,43]. This framework can help to understand decision-making and agent interactions related to adaptation and conflict. This is a modeling technique that examines the evolution of human actions and interactions in constrained dynamic systems. The framework (shown in Fig. 1) is comprised of five major components: Capability (K), Investments (C), Action paths (A), environmental states (X), and Values (V). In response to the environment, actors invest their capabilities in actions to reduce risks and increase net benefits. The investments may include capital, resources, and financial investments that can be allocated across multiple action pathways based on their priority. Action paths are the strategies by which actors increase their values and accomplish their goals. Actor investments influence the state of the environmental system, and the risk-benefit analysis measures the likelihood of conflict and the need for adaptation [31].

The VIABLE framework has been extensively used in agent-based modeling and system dynamics studies for understanding stability and conflict in socio-ecological systems. Scheffran [32] developed this approach to understand strategic stability in the context of the arms race [32,44,45] and then generalized it to analyze the stability and complexity of conflict, which is a dynamic interaction among agents driven into non-viable states and social learning to contain conflict potential to tolerable levels or transform it to cooperation. Later the model was expanded to understand environmental conflicts [[46], [47], [48], [49], [50], [51], [52], [53]]. Previous studies have utilized the VIABLE framework as a basis for assessing agent-based models in various fields, such as emission trading [52,54], fisheries [30], sustainable energy [53], flooding [55], as well as mobility [56,57].

The VIABLE framework has demonstrated its extensive interdisciplinary utility in multiple fields. These include the contestation dynamics of conflict studies, resource economics, energy transition, climate change and social-ecological agroecosystem [58]. Such an extensive reach of this framework with distant fields provides a testament to its adaptability and robustness in handling different systems, making it a prime candidate for this study. For the first time, we incorporate a statistical approach within this framework, introducing another novelty in the current study. We find that this innovative method significantly enhances our understanding of the complex linkages of capital adaptation relationship. We operationalize this in this study as farmers holding livelihood capital that serves as their capabilities. Farmers can invest in crops, land, or water to improve their capabilities, reduce risks, and increase benefits. The priorities for investing can vary based on the farmer's goals, which can include profit maximization, subsistence, social status, or competition with neighboring farmers. The actors face constraints that can limit their capability to invest. These constraints are classified as either human or natural. Additionally, we introduced factors such as climatic and non-climatic conditions that can influence farmers' decision-making and adaptation processes.

We chose the VIABLE framework for our study due to its exceptional capacity in dissecting how Pakistani farmers employ their sustainable livelihood capital to accommodate climate change adaptations in their agricultural practices. Despite encountering a variety of constraints, both natural and human-made, resulting from an interplay of climatic and non-climatic factors, farmers are often tasked with making decisions about the allocation of their capital towards land or water resources. The VIABLE framework's unique ability to assimilate these variables into a cohesive structure is what makes it fit for addressing our research questions.

## 3 Methodology

### 3.1 Study area

**Fig. 2:**
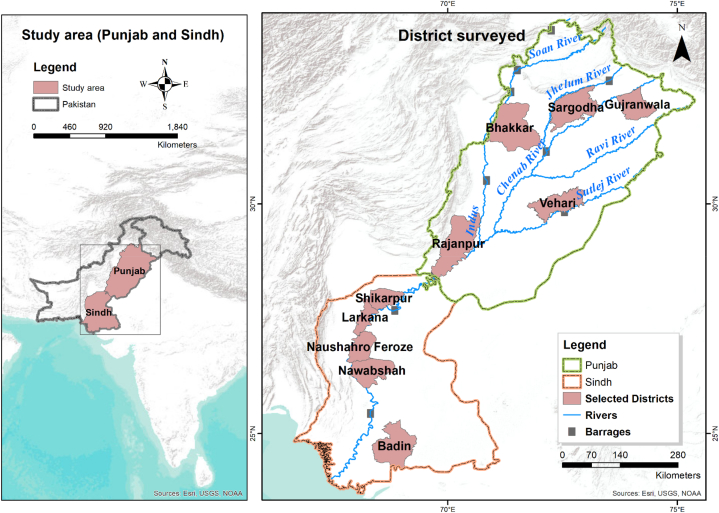
Map of the study area and data collection.

We conducted a field survey in the irrigated agricultural plains in the Indus basin (Fig. 2). The Indus Basin's irrigated agricultural plains are vital to Pakistan's economy and food security. The Indus River and its tributaries provide irrigation water to the basin's fertile lands through a vast network of canals and dams. This irrigation network supports cultivating numerous crops, including wheat, rice, sugarcane, cotton, and various fruits and vegetables. The area is famous for its highly productive agriculture due to the use of a relatively modern farming technique [59]. This area has a high number of small farmers who rely on farming for their livelihood, and they have experienced significant improvements in crop yields and productivity, which has increased food security. We conducted face-to-face interviews with small farmers in the irrigated agricultural regions of Punjab and Sindh provinces using a structured interview schedule. This area was chosen for our study due to its significant contribution to the country's agricultural output and its vulnerability to the impacts of climate change.

The study area spans 16.85 million hectares (Mha) and includes three major reservoirs, 12 inter-river link canals, and 44 main canals for irrigation control [59]. It possesses the world's largest irrigation system, with nearly 80 % of cultivated land irrigated (Muhammad et al., 2016), producing 90 % of the country's harvests (Zhu et al., 2013). The region represents about 40 % of Pakistan's total area and is home to 74 % of the country's population. The soil of these plains comprises alluvium deposits accumulated by the actions of the Indus River and its associated tributaries in the geological past. This soil property makes the area fertile for agricultural purposes. Pakistan is among the world's top ten producers of cotton, sugarcane, wheat, mango, dates, and Kinnow (citrus). The four dominating crops (rice, cotton, wheat, and sugarcane) contribute 4.9 % to Pakistan's gross domestic product. However, water resources in the region are highly stressed, whether judged by per capita water availability or by the ratio of withdrawals to runoff [60].

The mean average temperature in Punjab ranges from −2° to 45 °C, and exceptionally reaches 50 °C in summer and drops down to −8 °C in winter. In Sindh, temperatures rise above 46 °C from May to August and drop to 2 °C in winter. The interior of lower Sindh experienced up to 53.5 °C in 2010, the fourth-highest reading ever recorded in Asia [61,62]. Most regions in Punjab province receive moderate to high rainfall ranging from ∼275 to 830 mm/year, while Sindh province receives ∼150–180 mm/year. The region experiences a decrease in rainfall if we approach from north to south. Recent calculations in 2021 estimate a decreasing precipitation trend all around Pakistan with −1.11 mm/year [63].

The elevation of the Indus plain varies from 300 m in northern Punjab to 75 m near the southern border of Punjab, down to the Arabian Sea. In the plains, the slope fall rate is 0.3 m per 1.6 km [64]. The lower Indus Plain is part of Sindh province, the second largest province in population. Both provinces' areas are mainly agricultural, which is under stress due to the region's lack of rain and desertification trends.

### 3.2 Population and sampling

The distribution of farmland among Pakistani farmers is highly skewed. In Pakistan, 28 % of the land is cultivated by 80 % of the farmers. Pakistan has 7.4 million small farmers who own less than 12 acres (5 ha) of land [65]. In this study, we deal with small farmers (with landholdings of ≤16 acres) cultivating in irrigated Punjab and Sindh areas. These small farmers are spread across the entire 66 districts of Punjab and Sindh provinces. We used a multicriteria-based spatial cluster sampling strategy in various stages to select respondents from these districts.

In the first stage, we chose five districts from Punjab and five from Sindh with the help of their physiographic and irrigation maps. Punjab province has five rivers containing interfluve with distinct physiographic and soil characteristics. These interfluves are irrigated by Terbela and Mangal reservoirs, while Guddu, Sukkur, and Kotri irrigate the farmlands of Sindh province. We randomly chose one district from each interfluve in Punjab. We selected Bhakkar from Sindh Sagar doab, Sargodha from Chaj doab, Gujranwala from Rachna doab, and Rajanpur to represent the area out of the interfluve areas. Another criterion of selection was irrigation control of the Punjab plains. Bhakkar, Vehari, and Rajanpur take their irrigated water from the Terbela reservoir, while Sargodha is from the Mangla reservoir. Gujranwala is not controlled by any of the reservoirs directly. The Terbela reservoir irrigates Bhakkar, Vehari, and Rajanpur districts, while the Mangla reservoir irrigates Sargodha. Gujranwala is not controlled by any of the reservoirs directly.

For selecting districts from the Sindh province, we only considered their irrigation control because the irrigated land of Sindh is not physiographically diverse. As a result, we chose the districts of Shikarpur (next to the Guddu barrage), Badin (close to the Kotri barrage), Larkana, Naushahro Feroze, and Shaheed Benazirabad (near Sukkur barrage).

In stage two, we covered all Tehsils and Talukas (sub-unit of the district) in every district and visited 39 tehsils. In stage three, we randomly selected mauzas (the smallest revenue-collecting unit in Pakistan) based on the best spatial coverage of the Tehsil. In the last stage, we selected the respondents for the interview based on our convenient road connectivity to reach out to their households and farmland. Overall, 800 and precisely 80 farmers were interviewed from each district. We conducted a minimum of 10 and a maximum of 35 interviews with farmers from each Tehsil, with an overall target of 80 interviews per district. The number of Tehsils in each district is different, which varies the number of interviews in each Tehsil. We also noted the geographic coordinates of the farmlands of the respondents.

### 3.3 Development of scale and data collection

**Fig. 3:**
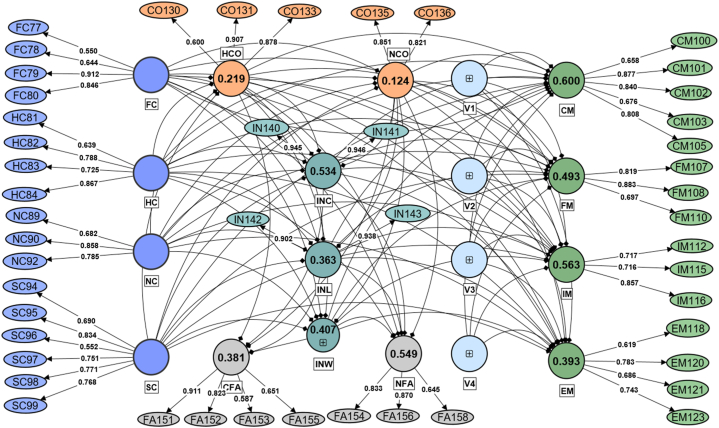
Measurement Model based on the VIABLE framework, Circles representing latent constructs with their R^2^ values inside.

We deconstructed the components of the VIABLE framework into a set of questions and statements asking about the agreement and disagreement of farmers. Previous studies [[66], [67], [68]] also used a similar approach to questionnaire development. We deconstructed farmers' capabilities, agricultural investment, farming purpose, factors, constraints, and adaptation. We itemized these constructs into a close-ended questionnaire (see S1 in Supplementary Materials) based on a five-point Likert scale with some open-ended basic demographic information about the respondents. Fig. 3 shows our respondents' education, farming experience, and secondary occupation. Other than the basic information of the respondents, we had 72 questions addressing the key components of the VIABLE model.

To represent the components of the VIABLE model, we subdivided the capabilities of farmers into Financial Capital (FC), Human Capital (HC), Natural Capital (NC), and Social Capital (SC). Our categories of farmers' capabilities are based on a sustainable livelihood framework that encompasses the factors that enable individuals to live [[69], [70], [71], [72]]. We categorized adaptation into four categories: Crop management (CM), Farm management (FM), Irrigation management (IM), and Economic Management (EM). We adapted these categories from the adaptation paradigm model of farmers [73] and grouped the constraints section into Human constraints (HCO) and Natural constraints (NCO), while factors of decision-making are classified as Climatic Factors (CF) and Non-climatic Factors (NF). In the field survey, we asked the respondents to rate each of these items on a five-point Likert scale ranging from strongly disagree to strongly agree. The scale contains a neutral option in the middle of disagreement and agreement. The Likert scale is a psychometric response scale in which respondents indicate their level of agreement with a statement ranging from strongly disagree (1), disagree (2), neutral (3), agree (4), and strongly agree (5) [74].

We started our data collection in December 2021 and completed it in March 2022 with the help of enumerators. Before field visits, we provided off-field and in-field training to the enumerators. We briefed them regarding the objective of our study and data collection methods. We conducted five online interviews with farmers in the Gujranwala district to pre-test the questionnaire. After these interviews, we rephrased the statements of some of the questions and added measurement units of area and distance in our demographic information section. We were able to obtain responses from a total of 913 farmers. However, we rejected 113 responses due to several quality concerns, such as double entries (27), incomplete submissions (19), respondents' misconduct (44), and readability issues (23). Finally, we were able to narrow the pool of surveys down to 800 useable responses.

### 3.4 Data analysis

To evaluate the research model, we employed PLS-SEM (Partial Least Squares Structural Equation Modeling) with the SmartPLS 4 software [75]. PLS-SEM is a statistical technique that combines factor and regression analysis to evaluate a model's relationship among variables [76]. It assesses the factor loadings, reliability, and validity of constructs, including the relationships among variables in a research model. This technique has established its predictive success in multiple studies [77]. In PLS-SEM, the emphasis is placed on discovering the combinations of variables that are most strongly associated with a particular latent construct instead of focusing on individual variables. This method is particularly useful when working with complex models [77]. Assessing a research model using PLS-SEM involves evaluating the measurement and structural models in two separate steps.

### 3.5 Measurement model

**Fig. 4:**
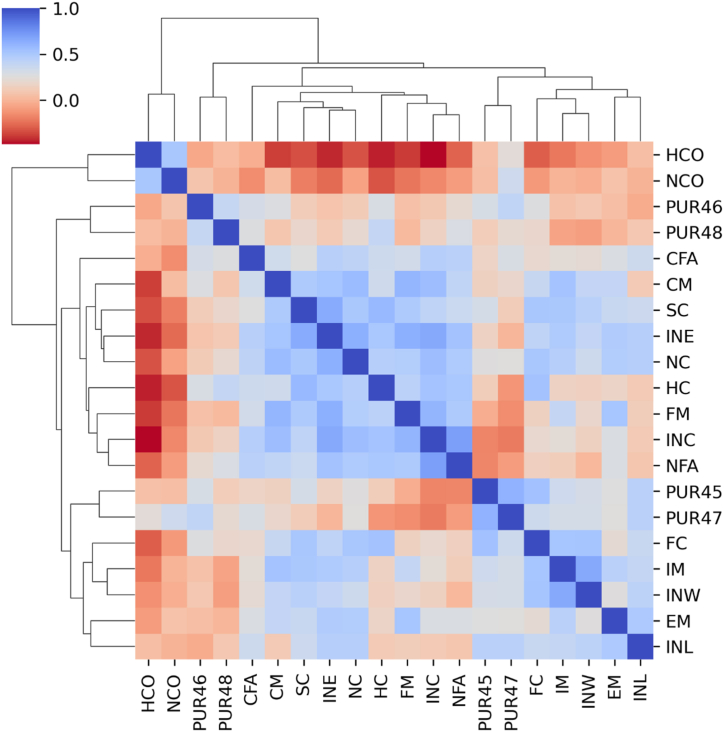
Cluster map of mutual correlations of latent variables used in the model.

**Fig. 5:**
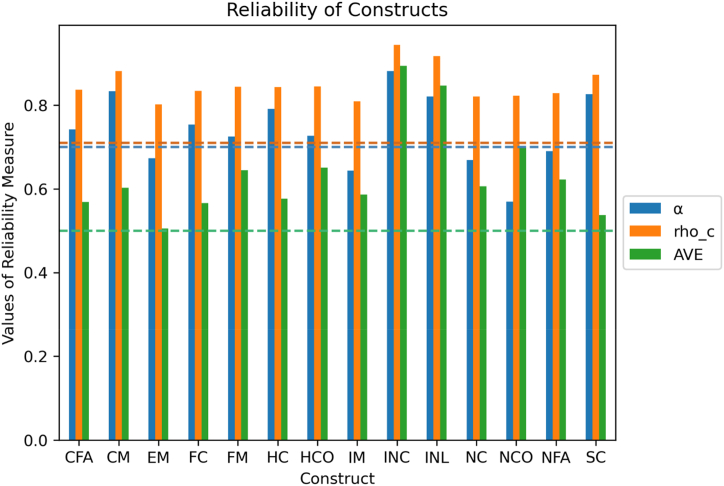
Reliability measures Cronbach's alpha, Composite reliability (rho_c), and Average variance extracted (AVE) of constructs with their respective cut of value lines.

The measurement model (Fig. 4) specifies the relationships between the latent and observed variables through factor loadings. Table 1 shows the details of latent and observed variables, while Table 2 contains the factor loadings of observed variables that we used in the model. These factor loadings represent the strength and direction of the relationships between the latent and observed variables [78]. The values of factor loadings help in defining latent constructs that are well correlated (see Fig. 5). For VIABLE-SEM, we measured 80 variables in the field with the questionnaire. 72 questions were addressing VIABLE framework. These 72 observed variables were approaching 19 first-order latent constructs. A group of items leads to a first-order latent construct which we computed based on the factor loading value of each item. PLS-SEM computes these values as Latent Variable Scores (LVS). Table 2 shows the factor loading details of our first-order latent variables. The first-order latent construct leads to second-order formative constructs which we used in our structural model in section 3 of this paper.

**Table 1:** Details of constructs and their codes.

Construct/Latent variables	Role of variable	Code	Items/Observed variables	Removed
**Capital**	Independent Variable	–		
Financial Capital		FC	77,78,79,80	–
Human Capital		HC	81,82,83,84	–
Natural Capital		NC	89,90,91,92	91
Social Capital		SC	93,94,95,96,97,98,99	93
**Adaptation**	Dependent Variable	**-**		**-**
Crop Management		CM	100,101,102,103,104, 105	104
Farm Management		FM	106,107,108,109,110	106,109
Irrigation Management		IM	111,112,113,114,115, 116,117	111,113,114,117
Economic Management		EM	118,119,120,121,122, 123, 124	119,122,124
**Constraints**	Mediators	**-**		**-**
Human Constraints		HCO	130,131,132,133,137	132,137
Natural Constraints		NCO	134,135,136	134
**Factors**	Mediator + Moderator	–		
Climatic Factors		CFA	151,152,153,155	–
Non-Climatic Factors		NFA	154,156,157,158,159	159,157
**Investment Priorities**	Mediators	–		
Crop Investment		INC	140,141	–
Land Investment		INL	142,143,144	143
Water Investment		INW	145,146	145

**Table 2:** Factor loadings.

Construct	Item code	Loadings	Construct	Item code	Loadings
Financial Capital	FC77	0.55	Crop Management	CM100	0.66
	FC78	0.64		CM101	0.88
	FC79	0.91		CM102	0.84
	FC80	0.85		CM103	0.68
Human Capital	HC81	0.64		CM105	0.81
	HC82	0.79	Farm Management	FM107	0.82
	HC83	0.73		FM108	0.88
	HC84	0.87		FM110	0.70
Natural Capital	NC89	0.68	Irrigation Management	IM112	0.72
	NC90	0.86		IM115	0.72
	NC92	0.79		IM116	0.86
Social Capital	SC94	0.69	Economic Management	EM118	0.62
	SC95	0.83		EM120	0.78
	SC96	0.55		EM121	0.69
	SC97	0.75		EM123	0.74
	SC98	0.77	Climatic Factors	FA151	0.91
	SC99	0.77		FA152	0.82
Human Constraints	CO130	0.60		FA153	0.59
	CO131	0.91		FA155	0.65
	CO133	0.88	Non-Climatic Factors	FA154	0.83
Natural Constraints	CO135	0.85		FA156	0.87
	CO136	0.82		FA158	0.65
Crop Investment	IN140	0.95	Competition	V1/PUR45	1.00
	IN141	0.95	Revenue Maximization	V2/PUR46	1.00
Land Investment	IN142	0.90	Social Status	V3/PUR47	1.00
	IN143	0.94	Subsistence	V4/PUR48	1.00
Water Investment	IN146	1.00

**Fig. 6:**
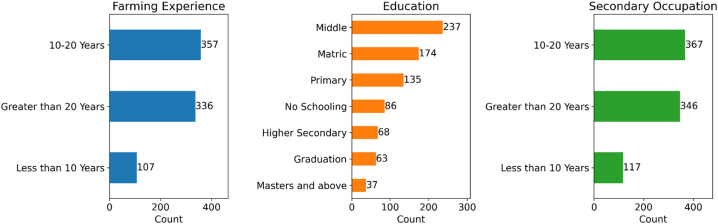
Socio-demographic traits of respondents.

The quality criteria for the measurement model are assessed through convergent and discriminant validity (see Supplementary Material S3). Convergent validity is assessed with factor loadings (≥0.70), Average Variance Extraction (AVE≥0.50) [79], and Composite reliability (≥0.70) [80]. Hence, all constructs in our model possess convergent validity (Fig. 6). AVE is calculated as the proportion of variance in an observed variable explained by the latent variable it is supposed to measure. If the AVE values are high (i.e., close to 1.0), the observed variables measure the same construct with high reliability. If the AVE values are low (i.e., close to 0.0), it indicates that the observed variables may be measuring different constructs or that the measure of the construct is unreliable [81]. Our model establishes a good range of the AVE values, as shown in Fig. 6**.**

Discriminant validity represents the distinctiveness of a variable or the degree to which each latent construct is distinct from the other construct in the model. This is a measure of the uncorrelatedness of variables in the model. This is typically done by examining the correlations between constructs and the cross-loadings of the manifest variables onto their respective constructs. In PLS-SEM, the measurement model is assessed through the Fronell-Larcker Criterion (F&L), Cross Loadings, and Heterotrait-monotrait Ratio of Correlations (HTMT). The most conservative threshold value of the HTMT ratio is less than or equal to 0.90. In this study, all the values of HTMT are less than the threshold value of 0.90 [79] (see Supplementary Material S3). Our model established the required quality criteria of discriminant validity. To achieve this validity criterion, we removed the indicators with lower factor loadings [82]. Out of 72 items, we removed 19 questions due to lower factor loadings (see Table 1, Table 2). After achieving the quality criteria of the measurement model, we were left with 53 items for analysis for the higher-order construct in our structural model.

Cluster map Fig. 5 is a graphical representation of a data matrix that uses hierarchical clustering to arrange the rows and columns of the matrix into clusters based on the similarity of their values. The Figure reveals the quality criteria of our constructs because the cluster map indicates the distinctiveness and mutual connection of the variables. In the map, we can identify different groups of constructs that are correlated to each other.

## 4 Results

### 4.1 Profile of respondents

Our respondents were farmers cultivating in the irrigated agricultural plains of Punjab and Sindh province. Fig. 3 shows the details of the basic information of our respondents. The respondents were aged 20–77 years. Many of the respondents (90 %) were educated, but only 12.5 % had higher education (Graduation and above), while the rest of the farmers (76.75 %) were up to a higher secondary level of education. Most of the farmers were experienced; only 13.3 % had less than ten years of experience in farming, while 44.6 % had 10–20 years of experience. All other farmers (42 %) had been farming for more than 20 years or had inherited farming from their ancestors. We also asked about the secondary occupation of farmers other than farming. We found that more than half of our respondents (57 %) are attempting to cover their living expenses through other means, such as running their businesses (26.7 %), working in the public sector (10 %), working in the private sector (8 %), and performing other odd jobs (14 %).

### 4.2 Structural model

**Fig. 7:**
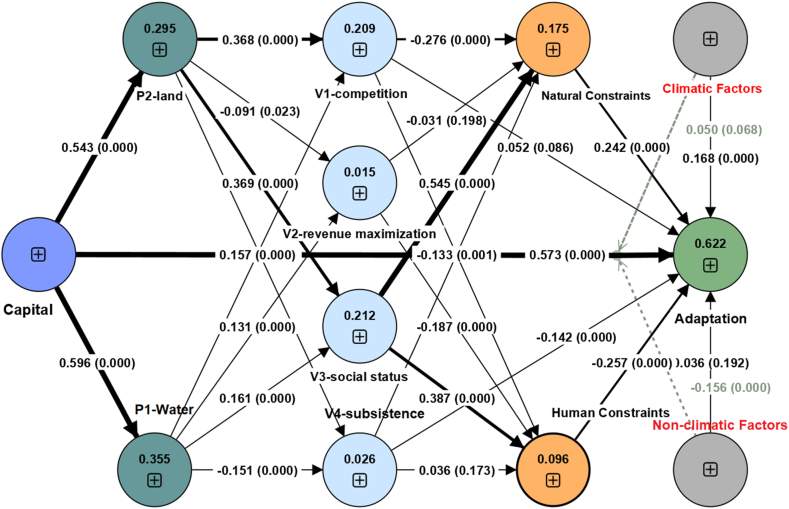
The VIABLE Structural Equation Model: Circles representing second-order constructs with their R^2^. Connecting lines represent relationships, annotated with β coefficients and p-values.

The structural model specifies the relationships between the latent and observed variables through a set of paths and their coefficients. Fig. 7 is the schematic display of our model which we termed VIABLE-SEM. The values shown on the connecting lines of variables are their path coefficients and their corresponding p values. These path coefficients represent the strength and direction of the relationships between the latent and observed variables [78,83]. The lines represent the interconnection of constructs in our model, while the width of each network represents the strengths of a connection. The breadth of lines or connections represents the magnitude of the path coefficient among the variables. The lines are more comprehensive, which had a more path coefficient, and the lesser the path coefficient, the lesser the breadth of the line.

**Fig. 8:**
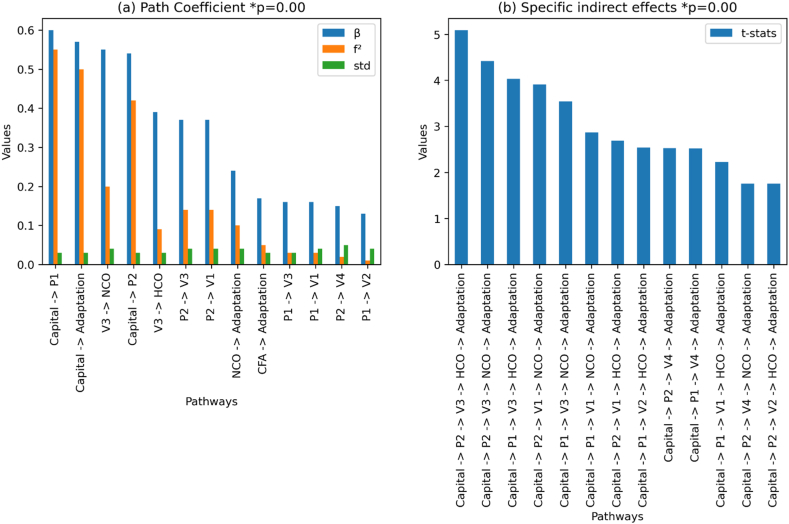
Highly significant pathways (models) based on total and specific indirect effects of the VIABLE-SEM.

Our hypothesis (H1) evaluates whether a significant model emerges by employing the VIABLE framework for assessing the role of farmers' livelihood capabilities in farming adaptation practices. We tested our hypotheses by employing mediation and moderation analyses in the model. We found multiple significant pathways by evaluating the model's total and specific indirect effects. Based on path coefficients of pathways in the model, we found “Capital” has a significant impact (total effect) on “Adaptation” (β = 0.573, t = 17.05, p = 0.00). Fig. 8 (a) and Table 3 show the 13 highly significant pathways in the model. We found that there is a stronger relationship between capital and adaptation, capital and P1 (investment in water, β = 0.60, f2 = 0.550, t = 23.50, p = 0.00), and Capital and P2 (Investment in Land, β = 0.54, t = 21.94, f2 = 0.418, p = 0.00). Hence, our H1 was supported.

**Table 3:** Path coefficients.

Relationships	β Coefficient	Effect size (f^2^)	std	stats	p-value
Capital → P1	0.60	0.550	0.03	23.50	0.00
Capital → Adaptation	0.57	0.503	0.03	17.05	0.00
V3 → NCO	0.55	0.202	0.04	12.37	0.00
Capital → P2	0.54	0.418	0.03	21.94	0.00
V3 → HCO	0.39	0.093	0.03	11.43	0.00
P2 → V3	0.37	0.142	0.04	10.21	0.00
P2 → V1	0.37	0.141	0.04	10.38	0.00
NCO → Adaptation	0.24	0.103	0.04	6.96	0.00
CFA → Adaptation	0.17	0.048	0.03	5.58	0.00
P1 → V3	0.16	0.027	0.03	5.11	0.00
P1 → V1	0.16	0.025	0.04	4.29	0.00
P2 → V4	0.15	0.020	0.05	3.30	0.00
P1 → V2	0.13	0.014	0.04	3.33	0.00

### 4.3 Quality criteria for structural model

In partial least squares structural equation modeling (PLS-SEM), R-squared (r^2^), F-squared (f^2^), and beta Coefficient (β) represents the quality of the model. Table 3 shows the values of r^2^, f^2,^ and β. R-squared is a statistical measure representing the proportion of variance in the dependent variable explained by the independent variables in a regression model (see Supplementary Material S4). It is calculated as the ratio of variance explained by the model to the total variance in the data. r^2^ ranges from 0 to 1, with higher values indicating a better fit of the model to the data. At the same time, F-Square is the change in R-Square when an exogenous variable is removed from the model.

Moreover, f-square is also called effect size, which is interpreted as small when it is≥0.02, medium for≥0.15, and≥0.35 for large [84]. F-square can be interpreted as a measure of the strength of the relationship between the dependent and independent variables. The results of our model show good values of f^2,^ which shows the strengths of relationships as shown in Fig. 8 (a) and Table 3.

Our model reveals that capital has the most significant effect on (P1) Investments in Water (f^2^ = 0.550), followed by adaptation (f^2^ = 0.503). The Social status (V3) effect on Natural constraints (NCO) (f^2^ = 0.202), Capital on (P2) Land related management (f^2^ = 0.418), (V3) Social status has an effect on (HCO) Human constraints (f^2^ = 0.093), (P2) land related management on (V3) Social status (f^2^ = 0.142), (P2) land related management on (V1) Competition (f^2^ = 0.141), (NCO) Natural constraints on Adaptation (f2 = 0.103), and Climatic factors (CFA) on Adaptation (f^2^ = 0.048).

### 4.4 Mediation analysis

Mediation analysis was performed to assess the mediating role of investment options (for land P1 and water P2), the purpose of farming (V1 competition, V2 social status, V3 profit maximization, and V4 subsistence), constraints (NCO natural constraints, HCO human constraints) and factors (CFA climatic factors and NFA non-climatic factors) affecting farming practice.

For further analysis of H1, we evaluated the specific indirect effects in the model (Fig. 8 (b)). The results of specific indirect effects also supported our H1. We found 11 pathways with high significance and good t statistics value. Fig. 8 (b) shows the pathways ranging from capital to investment options (i.e., P1 and P2) to the purpose of farming (i.e., V1, V2, V3, and V4). The values of t statistics for pathways with p = 0.00 range from 5.09 to 2.69 (see Table 3). We found that investment options and the purpose of farming mediate the relationship between Capital and Adaptation. The results show that the total effect (H1) was positive and significant (β = 0.573, t = 17.05, p = 0.00).

The results revealed a significant total effect (β = 0.591, t = 17.94, p = 0.00). When the mediators were introduced into the model, this effect was slightly decreased, and the direct relationship between Capital and Adaptation was still found to be significant (β = 0.573, t = 17.05, p = 0.00). Hence, this shows mediators' complementary partial mediation role in the relationship between Capital and Adaptation (See Fig. 7 and Table 4). Some mediators also showed competitive partial mediation with negative coefficients (see Fig. 6), but we are extending our analysis toward competitive partial mediation. Therefore, our H2 is supported.

**Table 4:** Mediation analysis through specific indirect effects.

Path	Path coefficient	std	t-statistics	p-values
Capital → P2 → V3 → NCO → Adaptation	0.03	0.01	4.42	0.00
Capital → P1 →V3 → NCO → Adaptation	0.01	0.00	3.54	0.00
Capital →P2 → V1 → HCO → Adaptation	0.01	0.00	2.69	0.00
Capital →P1 → V4 → Adaptation	0.01	0.01	2.52	0.01
Capital → P1 → V2 → HCO → Adaptation	0.00	0.00	2.54	0.01
Capital → P1 → V1→ HCO → Adaptation	0.00	0.00	2.23	0.01
Capital → P2 → V4 → NCO → Adaptation	0.00	0.00	1.76	0.04
Capital → P2 → V2 → HCO → Adaptation	0.00	0.00	1.76	0.04
Capital → P1 → V3 → HCO → Adaptation	−0.01	0.00	4.03	0.00
Capital → P2 → V1 → NCO → Adaptation	−0.01	0.00	3.91	0.00
Capital → P1→ V1 → NCO → Adaptation	−0.01	0.00	2.87	0.00
Capital → P2 → V4 → Adaptation	−0.01	0.01	2.53	0.01
Capital → P2 → V3 → HCO → Adaptation	−0.02	0.00	5.09	0.00

### 4.5 Path coefficient specific indirect effects

We stated H1: “Significant pathways emerge by taking farmers' livelihood capabilities as an independent variable, investment priorities, farming purpose and constraints as mediator and adaptation as an outcome.” Hence our H1 accepted that multiple significant pathways (ranges from p-value 0.00 to 0.01) emerge (see Table 4) by employing farmers' capital as independent and adaptation as a dependent variable with multiple mediators.

### 4.6 Moderating effect

The moderating effect refers to the influence of one variable (the moderator) on the relationship between two other variables [83]. A moderating effect occurs when the strength or direction of the relationship between the predictor and criterion variables varies depending on the level of the moderator variable [85,86].

The study assesses the moderating role of climatic factors and non-climatic factors on the positive relationship between capital and adaptation. Without including the moderating effect (NFA x Capital & CFA x capital), the R^2^ value for adaptation was 0.556. This shows that capital accounts for a 55 % change in adaptation. Including the first interaction term NFA x Capital, the R^2^ increased to 0.599. Furthermore, by introducing the second interaction term CFA x Capital, the R^2^ increased to 0.622, which shows an increase of 6.6 % in variance can be explained in the dependent variable (Adaptation) after introducing the moderators in the model.

Further, the significance of moderating effect was analyzed, and the results (β = -0.156, t = 5.456, p = 0.00) revealed (Table 5) a negative and highly significant moderating impact of NFA on the relationship between Capital and Adaptation. At the same time, there is a positive (β = 0.050, t = 1.494, p = 0.068) and weakly significant moderating effect of CFA on the relationship between Capital and Adaptation. This result shows that the relationship between capital and adaptation strengthens with increased NFA. With the rise in CFA, the relationship between capital and adaptation weakens. Hence our H3 is accepted as both factors are moderating significantly, but both types of factors are moderating oppositely.

**Table 5:** Moderation analysis.

Relationship	β Coefficient	SE	t-statistics	p-value
Moderating effect (NFA x Capital) →Adaptation	−0.156	0.029	5.456	0.000
Moderating effect (CFA x Capital) →Adaptation	0.050	0.033	1.494	0.068
Capital → Adaptation	0.573	0.034	17.050	0.000
CFA → Adaptation	0.168	0.030	5.575	0.000
NFA → Adaptation	0.036	0.042	0.872	0.192

**Fig. 9:**
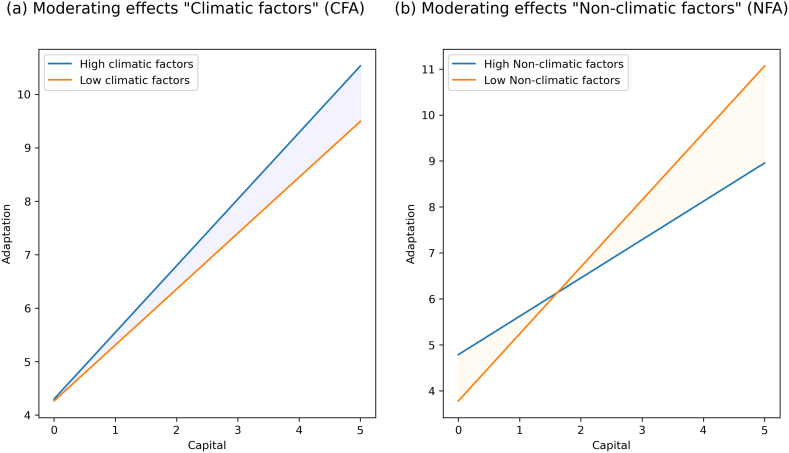
Simple slope analysis explaining moderating effects of Climatic and Non-climatic factors on the Capital-adaptation relationship.

Further, slope analysis is presented to understand the moderating effects (Fig. 9). As shown in Fig. 9 (b), the line is much steeper for low NFA; this indicates that at low NFA, the impact of capital on adaptation is much more robust compared to high NFA. In other words, if we increase capital, adaptation will increase. However, as shown in Fig. 9 (a), at higher CFA and lower CFA, the adaptation does not show much difference. In conclusion, with lower CFA, lower adaptation, and higher CFA, the adaptation is also slightly higher.

According to the Cohen [84] f-square criteria, the effect is small when it is≥0.02, medium for≥0.15, and≥0.35 for large. Therefore, the f-square for CFA x Capital → Adaptation is insignificant, while for NFA x Capital → Adaptation, the effect size is 0.043, which is very small but significant (at p = 0.003).

A negative interaction effect suggests that the relationship between NFA and the dependent variable is weaker when capital is high than when capital is low.

## 5 Discussion

This study investigates the relationship between livelihood capital and climate change adaptation in the Indus Plains' irrigated agricultural regions. It also examines how this relationship is affected by investment priorities, farming constraints, and various climatic and non-climatic factors. The findings of our model suggest that capital has the most significant and influential role in the farmers' adaptation strategies. All other variables, including investment options, farming purposes, and constraints, are less important than capital. This explains two-thirds of the observed variance in adaptation. The capital alone explains up to 57 % of the adaptation variance. Sargani et al. [87,88] reported similar findings in their study conducted in Sindh province. Our results are consistent with those reported in previous studies conducted in developing countries and align with those from other studies in neighboring countries of Pakistan, such as Nepal [89], China [90,91], and Iran [29].

Our model determined thirteen statistically significant adaptation pathways. These pathways explain the influence of livelihood capital with the mediating role of investment priorities, constraints, and purpose of farming in the adaptation process, which supports our mediation hypothesis. The role of capital is important in intermediate actions other than adaptation alone. Our model suggests that farmers' investments in water and land resources are equally significant. Furthermore, maintaining social status was the primary motivation for many farmers, rather than subsistence, profit maximization, or competition. The impact of natural constraints is stronger than human constraints, but both are highly significant. Our findings are consistent with Abid et al. [92] who reported that natural constraints like water scarcity were significant in farming in the Punjab region. According to this study, irrigated water is insufficient to fulfill crop requirements and maximize productivity. By contrast, some studies identified resource limitation as a barrier to adaptation [[93], [94], [95]].

The moderation analysis of our model revealed that climatic and non-climatic factors significantly influence the capital and adaptation relationship. This reveals that in the presence of climatic and non-climatic factors, a higher amount of capital can no longer help in achieving a higher adaptation rate. We expected this and stated it as our moderation hypothesis in the beginning. Chandio et al. [96] recently reported that climatic and non-climatic factors significantly influence agricultural adaptation in neighboring India, where farming practices in the plain areas are like the agriculture in the plain areas of Pakistan.

Our results revealed that the rise in climatic factors slightly increases the chances of adaptation, which means that a hostile climate can push farmers to take adaptive action. The rise in non-climatic factors lessens the adaptation despite having enough capital. This can be explained because non-climatic factors are similar to variables that contribute to farmers' capital; higher non-climatic factors mean a lower amount of capital.

The study found that non-climatic factors negatively influence the relationship between capital and adaptation with high significance, while climatic factors influence positively but with weak significance. The results further suggest that the effect of capital on adaptation increases in the absence of climatic and non-climatic factors. Without including factors, the overall model accounts for only 55 % of the variance in adaptation. However, when non-climatic factors are introduced as a moderator, this variance increases to 60 %, increasing to 62 % with the inclusion of climatic factors in the model. This suggests that farmers adapt more effectively in the presence of climatic and non-climatic factors. We propose the same in our third hypothesis that climatic and non-climatic factors moderate the relationship between capital and adaptation, supported by findings. However, it is essential to note that both factors are moderating oppositely.

Despite this valuable insight, it is important to consider the limitations of this study. Conducting fieldwork during the COVID-19 pandemic posed significant challenges. Gathering data from 800 farmers while adhering to safety protocols was a significant task and may have influenced the data collection process. Having focus on the irrigated agricultural regions in Pakistan means that our findings may not universally represent the diverse socio-economic and geographical realities of all Pakistani farmers. The use of structural equation modeling, with its inherent assumption of statistical linearity among variables, might not entirely capture the complex, non-linear relationships that often exist in real-world scenarios. Furthermore, the skewed distribution of farmland in Pakistan, despite our multicriteria-based spatial cluster sampling, might lead to underrepresentation of certain farmer groups. Lastly, the theoretical basis of the VIABLE framework, while effective, might not account fully for the varied and unpredictable nature of human responses to climate change. These limitations, while providing a realistic view of our study's constraints, also open avenues for further research. Future work could focus on broader sampling, incorporation of non-linear relationships, understanding collective decision-making influences, and factoring in the unpredictability of human behavior.

## 6 Conclusion

In conclusion, our study explores the relationship between livelihood capital and climate change adaptation with the mediating and moderating variables in irrigated agricultural regions of the Indus plain. Capital is the most significant factor in farmers' adaptation strategies in the Indus plain. Other variables such as water investment, land investment, farming purposes, and farming constraints are less important than capital. Investments in land and water are equally important in farmers' eyes when they make decisions about their investment options. Our research found that maintaining social status emerged as a primary motivation for farming among farmers in our study area. Our model explains approximately two-thirds of the adaptation process, while capital alone accounts for 57 %. The model identified 13 statistically significant pathways which explain the role of different mediators in the adaptation process. The study also found that the relationship between capital and adaptation is more significant without mediators. The moderation results suggest that climatic and non-climatic factors significantly influence the relationship between capital and adaptation. Non-climatic factors hinder the adaptation process, while climatic factors play a positive but weak role in the adaptation process. The results suggest that farmers adapt more effectively in the presence of these factors. The climatic and non-climatic factors are responsible for increasing the adaptive capacity of farmers. Overall, our findings are consistent with previous studies conducted in developing countries and neighboring countries of Pakistan. Our study helps us learn more about the complex relationship between capital, investments, constraints, farming purpose, factors, and adaptation. It also gives policymakers and people who work in agriculture and rural development useful information. It also gives policymakers and people who work in agriculture and rural development useful information.

## Funding statement

This study was in part supported by the Deutsche Forschungsgemeinschaft (10.13039/501100001659DFG, German Research Foundation) under Germany‘s Excellence Strategy—EXC 2037 ‘CLICCS–Climate, Climatic Change, and Society’ Project Number: 390683824, contributing to the Center for Earth System Research and Sustainability (CEN) of 10.13039/501100023890Universität Hamburg.

## Data availability statement

Data will be made available on request.

## CRediT authorship contribution statement

**Muhammad Mobeen:** Conceptualization, Formal analysis, Funding acquisition, Investigation, Methodology, Project administration, Software, Validation, Visualization, Writing – original draft, Writing – review & editing. **Khondokar Humayun Kabir:** Conceptualization, Data curation, Funding acquisition, Methodology, Resources, Writing – original draft, Writing – review & editing. **Uwe Schneider:** Conceptualization, Funding acquisition, Investigation, Project administration, Resources, Supervision, Writing – original draft, Writing – review & editing. **Tauqeer Ahmed:** Data curation, Investigation, Resources, Validation, Writing – original draft, Writing – review & editing. **Jürgen Scheffran:** Conceptualization, Funding acquisition, Investigation, Methodology, Project administration, Resources, Supervision, Writing – original draft, Writing – review & editing.

## Declaration of competing interest

The authors declare that they have no known competing financial interests or personal relationships that could have appeared to influence the work reported in this paper.

## References

[1] Eckstein D. (2019).

[2] Kreft S., Eckstein D., Melchior I. (2013).

[3] Wheeler T., von Braun J. (2013). Climate change impacts on global food security. Science.

[4] Abid M. (2015). Farmers' perceptions of and adaptation strategies to climate change and their determinants: the case of Punjab province, Pakistan. Earth Syst. Dynam..

[5] Schilling J. (2013). Vulnerability to environmental risks and effects on community resilience in mid-west Nepal and south-east Pakistan. Environ. Nat. Resour. Res..

[6] Ahmed M., Schmitz M. (2011). Economic assessment of the impact of climate change on the agriculture of Pakistan. Bus. Econ. Horiz..

[7] Osbahr H. (2010). Evaluating successful livelihood adaptation to climate variability and change in southern Africa. Ecol. Soc..

[8] Abid M., Schneider U.A., Scheffran J. (2016). Adaptation to climate change and its impacts on food productivity and crop income: perspectives of farmers in rural Pakistan. J. Rural Stud..

[9] Jezeer R.E. (2019). Influence of livelihood assets, experienced shocks and perceived risks on smallholder coffee farming practices in Peru. J. Environ. Manag..

[10] Khanal U. (2018). Climate change adaptation strategies and food productivity in Nepal: a counterfactual analysis. Climatic Change.

[11] Pandey R. (2017). Agroecology as a climate change adaptation strategy for smallholders of tehri-Garhwal in the Indian Himalayan region. Small-scale Forestry.

[12] Adger W.N., Arnell N.W., Tompkins E.L. (2005). Successful adaptation to climate change across scales. Global Environ. Change.

[13] Evans L.S. (2016). Structural and psycho-social limits to climate change adaptation in the Great barrier reef region. PLoS One.

[14] Deressa T.T., Hassan R.M., Ringler C. (2011). Perception of and adaptation to climate change by farmers in the Nile basin of Ethiopia. J. Agric. Sci..

[15] Deressa T.T. (2009). Determinants of farmers' choice of adaptation methods to climate change in the Nile Basin of Ethiopia. Global Environ. Change.

[16] Kato E. (2011). Soil and water conservation technologies: a buffer against production risk in the face of climate change? Insights from the Nile basin in Ethiopia. Agric. Econ..

[17] Bryan E. (2013). Adapting agriculture to climate change in Kenya: household strategies and determinants. J. Environ. Manag..

[18] Seo S.N., Mendelsohn R. (2008). An analysis of crop choice: adapting to climate change in South American farms. Ecol. Econ..

[19] Ali A., Abdulai A. (2010). The adoption of genetically modified cotton and poverty reduction in Pakistan. J. Agric. Econ..

[20] Schlenker W., Lobell D.B. (2010). Robust negative impacts of climate change on African agriculture. Environ. Res. Lett..

[21] Bradshaw B., Dolan H., Smit B. (2004). Farm-level adaptation to climatic variability and change: crop diversification in the Canadian prairies. Climatic Change.

[22] Esteve P., Varela-Ortega C., Downing T.E. (2018). A stakeholder-based assessment of barriers to climate change adaptation in a water-scarce basin in Spain. Reg. Environ. Change.

[23] Bastakoti R.C. (2014). Climate risks and adaptation strategies in the Lower Mekong River basin. Reg. Environ. Change.

[24] Dfid U. (1999).

[25] Ellis F. (2000).

[26] Baffoe G., Matsuda H. (2018). An empirical assessment of rural livelihood assets from gender perspective: evidence from Ghana. Sustain. Sci..

[27] Bryan B.A. (2015). What actually confers adaptive capacity? Insights from agro-climatic vulnerability of Australian wheat. PLoS One.

[28] Wu Z., Li B., Hou Y. (2017). Adaptive choice of livelihood patterns in rural households in a farm-pastoral zone: a case study in Jungar, Inner Mongolia. Land Use Pol..

[29] Dehghani Pour M. (2018). Revealing the role of livelihood assets in livelihood strategies: towards enhancing conservation and livelihood development in the Hara Biosphere Reserve, Iran. Ecol. Indicat..

[30] BenDor T., Scheffran J., Hannon B. (2009). Ecological and economic sustainability in fishery management: a multi-agent model for understanding competition and cooperation. Ecol. Econ..

[31] BenDor T.K., Scheffran J. (2019). Agent-based Modeling of Environmental Conflict and Cooperation.

[32] Scheffran J. (1989).

[33] Pretty J., Ward H. (2001). Social capital and the environment. World Dev..

[34] McDowell J.Z., Hess J.J. (2012). Accessing adaptation: multiple stressors on livelihoods in the Bolivian highlands under a changing climate. Global Environ. Change.

[35] Malek K. (2018). When should irrigators invest in more water-efficient technologies as an adaptation to climate change?. Water Resour. Res..

[36] Li L. (2023). Effects of social capital on farmers' choices of climate change adaptation behavior in Dazu District, China. Clim. Dev..

[37] Saptutyningsih E., Dewi Nurcahyani F. (2022). Is social capital important in coping with climate change? A case of agriculture sector in Gunungkidul, Indonesia. E3S Web Conf..

[38] Lobell D.B. (2008). Prioritizing climate change adaptation needs for food security in 2030. Science.

[39] Okada M. (2015). Modeling irrigation-based climate change adaptation in agriculture: model development and evaluation in Northeast China. J. Adv. Model. Earth Syst..

[40] Ozor N. (2011). Barriers to climate change adaptation among farming households of southern Nigeria. The Journal of Agricultural Extension.

[41] Karki S., Burton P., Mackey B. (2020). Climate change adaptation by subsistence and smallholder farmers: insights from three agro-ecological regions of Nepal. Cogent Social Sciences.

[42] Saint-Pierre P. (2011).

[43] Aubin J.-P., Saint-Pierre P. (2007). An introduction to viability theory and management of renewable resources. Advanced Methods for Decision Making and Risk Management in Sustainability Science.

[44] Jathe M., Krabs W., Scheffran J. (1997). Control and Game-theoretical treatment of a cost-security model for disarmament. Math. Methods Appl. Sci..

[45] Scheffran J., Huber R., Avenhaus R. (1996). Models for security policy in the post-cold war era.

[46] Scheffran J., Jathe M. (1996). Modelling the impact of the Greenhouse effect on international stability. IFAC Proc..

[47] Eisenack K., Scheffran J., Kropp J. (2006). Viability analysis of management frameworks for fisheries. Environ. Model. Assess..

[48] Link P.M. (2012). On foes and flows : vulnerabilities, adaptive capacities and transboundary relations in the nile river basin in times of climate change. L'Europe en Formation.

[49] Scheffran J. (2000). The dynamic interaction between economy and ecology. Math. Comput. Simulat..

[50] Scheffran J., Treitz M. (2004). Entscheidungstheorie und -praxis in industrieller Produktion und Umweltforschung.

[51] Scheffran J., Bendor T. (2009). Bioenergy and land use: a spatial-agent dynamic model of energy crop production in Illinois. Int. J. Environ. Pollut..

[52] Scheffran J., Leimbach M., Antes R., Hansjürgens B., Letmathe P. (2006). Emissions trading and business.

[53] Shaaban M. (2019). A dynamic sustainability analysis of energy landscapes in Egypt: a spatial agent-based model combined with multi-criteria decision analysis. J. Artif. Soc. Soc. Simulat..

[54] Scheffran J. (2002). Operations Research Proceedings 2001.

[55] Hokamp S., Rühe S., Scheffran J. (2020).

[56] Rodriguez-Lopez J.M. (2021). Technological and social networks of a pastoralist artificial society: agent-based modeling of mobility patterns. Journal of Computational Social Science.

[57] Peng Y. (2023). Simulating exposure-related human mobility behavior at the neighborhood-level under COVID-19 in Porto Alegre, Brazil. Cities.

[58] Shaaban M. (2023). Viability of the social–ecological agroecosystem (ViSA). SoftwareX.

[59] Steenbergen F., Basharat M., Lashari B. (2015). Key challenges and opportunities for conjunctive management of surface and Groundwater in mega-irrigation systems. Resources.

[60] Archer D.R. (2010). Sustainability of water resources management in the Indus Basin under changing climatic and socio economic conditions. Hydrol. Earth Syst. Sci..

[61] Huber D., Gulledge J. (2011).

[62] Abbas F. (2018). Prevailing trends of climatic extremes across Indus-Delta of Sindh-Pakistan. Theor. Appl. Climatol..

[63] Ali G. (2021). Spatial–temporal characterization of rainfall in Pakistan during the past half-century (1961–2020). Sci. Rep..

[64] Khan F.K. (2016).

[65] Naseer A. (2016). Current status and key trends in agricultural land holding and distribution in Punjab, Pakistan: implications for food security. J. Agric. Stud..

[66] Bhalerao A.K. (2022). Sustainable agriculture in Northeastern India: how do tribal farmers perceive and respond to climate change?. Int. J. Sustain. Dev. World Ecol..

[67] Bhalerao A.K., Rasche L., Schneider U.A. (2021). Preparing for a better future: delphi forecasts on competency development to enhance climate-resilient farming in Northeastern India. Int. J. Sustain. Dev. World Ecol..

[68] Abid M. (2016). Climate change vulnerability, adaptation and risk perceptions at farm level in Punjab, Pakistan. Sci. Total Environ..

[69] Reed M.S. (2013). Combining analytical frameworks to assess livelihood vulnerability to climate change and analyse adaptation options. Ecol. Econ..

[70] Serrat O., Serrat O. (2017). Knowledge Solutions: Tools, Methods, and Approaches to Drive Organizational Performance.

[71] Natarajan N. (2022). A sustainable livelihoods framework for the 21st century. World Dev..

[72] Chambers R., Conway G. (1992).

[73] Zobeidi T., Yaghoubi J., Yazdanpanah M. (2022). Developing a paradigm model for the analysis of farmers' adaptation to water scarcity. Environ. Dev. Sustain..

[74] Robinson J., Michalos A.C. (2014). Encyclopedia of Quality of Life and Well-Being Research.

[75] Sarstedt M., Cheah J.-H. (2019). Partial least squares structural equation modeling using SmartPLS: a software review. Journal of Marketing Analytics.

[76] Khan G.F. (2019). Methodological research on partial least squares structural equation modeling (PLS-SEM). Internet Res..

[77] Akter S., Fosso Wamba S., Dewan S. (2017). Why PLS-SEM is suitable for complex modelling? An empirical illustration in big data analytics quality. Prod. Plann. Control.

[78] Hair J. (2022).

[79] Henseler J., Ringle C.M., Sarstedt M. (2015). A new criterion for assessing discriminant validity in variance-based structural equation modeling. J. Acad. Market. Sci..

[80] Ringle C.M. (2020). Partial least squares structural equation modeling in HRM research. Int. J. Hum. Resour. Manag..

[81] Wetzels M., Odekerken-Schröder G., Van Oppen C. (2009). Using PLS path modeling for assessing hierarchical construct models: Guidelines and empirical illustration. MIS Q..

[82] Gefen D., Straub D. (2005). A practical guide to factorial validity using PLS-Graph: tutorial and annotated example. Commun. Assoc. Inf. Syst..

[83] Hair J.F. (2017).

[84] Cohen J. (1988).

[85] Dawson J.F. (2014). Moderation in management research: what, why, when, and how. J. Bus. Psychol..

[86] Dawson J.F., Richter A.W. (2006). Probing three-way interactions in moderated multiple regression: development and application of a slope difference test. J. Appl. Psychol..

[87] Sargani G.R. (2022). Impacts of livelihood assets on adaptation strategies in response to climate change: evidence from Pakistan. Environ. Dev. Sustain..

[88] Sargani G.R. (2021). How do gender disparities in entrepreneurial aspirations emerge in Pakistan? An approach to mediation and multi-group analysis. PLoS One.

[89] Adhikari B., Di Falco S., Lovett J.C. (2004). Household characteristics and forest dependency: evidence from common property forest management in Nepal. Ecol. Econ..

[90] Kuang F. (2020). Farmers' livelihood risks, livelihood assets and adaptation strategies in Rugao City, China. J. Environ. Manag..

[91] Kuang F. (2019). Influence of livelihood capital on adaptation strategies: evidence from rural households in Wushen Banner, China. Land Use Pol..

[92] Abid M. (2016). Climate change vulnerability, adaptation and risk perceptions at farm level in Punjab, Pakistan. Sci. Total Environ..

[93] Saddique N. (2022). A systematic review on farmers' adaptation strategies in Pakistan toward climate change. Atmosphere.

[94] Mahmood N. (2019). Wheat yield response to input and socioeconomic factors under changing climate: evidence from rainfed environments of Pakistan. Sci. Total Environ..

[95] Shahid R. (2021). Determinants of reactive adaptations to climate change in semi-arid region of Pakistan. J. Arid Environ..

[96] Chandio A.A. (2022). Modeling the impact of climatic and non-climatic factors on cereal production: evidence from Indian agricultural sector. Environ. Sci. Pollut. Control Ser..

