# Supplementary material for: Sustainable livelihood capital and climate change adaptation in Pakistan's agriculture: Structural equation modeling analysis in the VIABLE framework
Source: Heliyon. 2023 Oct 13;9(11):e20818. doi: 10.1016/j.heliyon.2023.e20818 (PMC10623177; doi:10.1016/j.heliyon.2023.e20818)
Supplement: Multimedia component 1 [file mmc1.docx]

**S1 Questionnaire**

**Famers’ Capabilities and their climate change adaptation strategies in irrigated farmland in Sindh and Punjab, Pakistan.**

|  | **Questions** | **Answers** | |
| --- | --- | --- | --- |
| 1 | Location (Latitude/Longitude) | Y or Lat: | X or Long: |
| 2 | Date of survey |  | |
| 3 | Name and contact of respondent |  | |
| 4 | Age of the respondent |  | |
| 5 | District, Tehsil, Village (Chak no. etc) |  | |

**Socio-demographic profile of respondents**

| 17 | Education  ① No schooling, ② Primary, ③ Middle ④ Matric  ⑤ HSSC, ⑥ Graduation, ⑦ Masters and above | ①②③④  ⑤⑥⑦ |
| --- | --- | --- |
| 19 | Farming experience in years ① less than 10, ② 10-20, ③ Above 20 | ①②③ |
| 20 | Secondary occupation other than farming ① Public employment, ② Private employment ③ Own business ④ Others ⑤ None | ①②③④⑤ |

**Part-2 VIABLE Model**

**Farming Purpose**

|  | **Tick the option that applies to you, Mark**① for Strongly Disagree, ② for Disagree, ③ for Neutral, ④ for Agree, ⑤ for Strongly agree | **Answers** |
| --- | --- | --- |
|  | **Purpose of farming (45-48)** |  |
| 45 | The purpose of my farming is to compete with other farmers | ①②③④⑤ |
| 46 | The purpose of my farming is revenue maximization | ①②③④⑤ |
| 47 | The purpose of my farming is to raise my social status | ①②③④⑤ |
| 48 | The purpose of my farming is subsistence (meeting the daily livelihood) | ①②③④⑤ |

**Capital of the farmer**

|  | **Statement** | **Answers** |
| --- | --- | --- |
|  | Tick the option that applies to you, Mark  ① for Strongly Disagree, ② for Disagree, ③ for Neutral, ④ for Agree, ⑤ for Strongly agree |  |
|  | **Financial capital** |  |
| 77 | I have an adequate size of my farmland | ①②③④⑤ |
| 78 | I have sufficient livestock at my farm | ①②③④⑤ |
| 79 | I have all the machinery needed for farming | ①②③④⑤ |
| 80 | I have sufficient capital at hand for the next year’s investment | ①②③④⑤ |
|  | **Human capital** |  |
| 81 | I have an adequate number of labours at my farm | ①②③④⑤ |
| 82 | My labour has good farming skills | ①②③④⑤ |
| 83 | My farmworkers have good physical fitness | ①②③④⑤ |
| 84 | I have good knowledge about agriculture | ①②③④⑤ |
|  | **Capital of the farmer**  **Natural capital** |  |
| 89 | The level of fertility of my farm is good | ①②③④⑤ |
| 90 | I am farming close to transportation networks (roads, rail etc.) | ①②③④⑤ |
| 91 | My farmland can support multiple crops | ①②③④⑤ |
| 92 | My farm is closer to the irrigation channel | ①②③④⑤ |
|  | **Social capital** |  |
| 93 | I have a good relationship with my neighbouring farmer | ①②③④⑤ |
| 94 | I have good relations with farmers’ associations | ①②③④⑤ |
| 95 | I have good networking and links to all experts of farming in my area | ①②③④⑤ |
| 96 | I trust in my social connections for solving my problems | ①②③④⑤ |
| 97 | I trust in the government agricultural institutions for solving my problems | ①②③④⑤ |
| 98 | I trust in the non-governmental organizations (NGOs) working for farmers | ①②③④⑤ |
| 99 | I trust in my networking about the farming community for my problem solving | ①②③④⑤ |

**Adaptation actions**

|  | **Statements** | Answers |
| --- | --- | --- |
|  | What is your take on the given adaptation practices under the changing climate and water conflicts? Mark  ① I am not aware of this practice  ② I know about this practice but I am not applying it  ③ I know about this practice and applying it  ④ I know about this practice and have a plan to apply in future  ⑤ I know about this practice and do not have a plan to apply in future |  |
|  | **Crop Management** |  |
| 100 | Cultivation of early cultivars | ①②③④⑤ |
| 101 | Cultivation of drought and water scarcity tolerant crops | ①②③④⑤ |
| 102 | Cultivation of salt-tolerant crops | ①②③④⑤ |
| 103 | Cultivation of crops that can produce more revenue | ①②③④⑤ |
| 104 | Adding tree plantations with the main crop (Agroforestry) | ①②③④⑤ |
| 105 | Cultivating legume cropping (Soybean, Chickpea etc.) | ①②③④⑤ |
|  | **Farm Management** |  |
| 106 | Changing the methods and techniques of cultivation | ①②③④⑤ |
| 107 | Re-sheduling the land preparation | ①②③④⑤ |
| 108 | Changing the fertilizers | ①②③④⑤ |
| 109 | Tree plantation | ①②③④⑤ |
| 110 | Modification of tillage system | ①②③④⑤ |
|  | **Irrigation Management** |  |
| 111 | Irrigation re-scheduling | ①②③④⑤ |
| 112 | Change method of irrigation (Shifting to drip, sprinkle irrigation) | ①②③④⑤ |
| 113 | Cementation of watercourse | ①②③④⑤ |
| 114 | Canal dredging or canal clearing | ①②③④⑤ |
| 115 | Modification of water allocation rules between individual farmers | ①②③④⑤ |
| 116 | Rainwater harvesting for future irrigation | ①②③④⑤ |
| 117 | New tube-well installation |  |
|  | **Economic Management** |  |
| 118 | Addition of livestock | ①②③④⑤ |
| 119 | Reduction of livestock | ①②③④⑤ |
| 120 | Migrating to the urban centre | ①②③④⑤ |
| 121 | Land renting | ①②③④⑤ |
| 122 | Land selling | ①②③④⑤ |
| 123 | Getting loans from banks | ①②③④⑤ |
| 124 | Change in number of farmworkers | ①②③④⑤ |
|  | **Social Network and Knowledge management** |  |
| 125 | Use of meteorological information | ①②③④⑤ |
| 126 | Taking advisory from the agricultural department | ①②③④⑤ |
| 127 | Contacting and talking with other farmers | ①②③④⑤ |
| 128 | Using local knowledge and wisdom/ taking advice from elder farmers sages etc. | ①②③④⑤ |
| 129 | Using TV or newspaper for taking information for farming | ①②③④⑤ |
|  | **Constraints:** To what extent are the following factors responsible for not changing your farming practice during the last ten years. Mark  ① for **Not at all**, ② for **Low,** ③ for **Medium,** ④ for **High,** ⑤ for **Very high** |  |
| 130 | Lack of money | ①②③④⑤ |
| 131 | Lack of information | ①②③④⑤ |
| 132 | Lack of motivation | ①②③④⑤ |
| 133 | Lack of farming skills | ①②③④⑤ |
| 134 | Water scarcity | ①②③④⑤ |
| 135 | Poor soil fertility | ①②③④⑤ |
| 136 | Insufficient size of land | ①②③④⑤ |
| 137 | Lack of manpower needed for making any change | ①②③④⑤ |

**Priorities of Investment options**

|  | **Statement** | Answers |
| --- | --- | --- |
|  | The answer is Yes or No ①for Yes for ②No |  |
| 138 | Do you ask for help from your neighbouring farmer if your harvest fails? | ①② |
| 139 | Do you get into conflict with your neighbouring farmer if your harvest fails? | ①② |
|  | If you get profit from your crop, how would you allocate it?  Rate the amount of investment from ① to ⑤, ① for the lowest and ⑤ for the highest |  |
| 140 | Investment in changing in cultivating a more (climatologically) suitable crop | ①②③④⑤ |
| 141 | Investment in expanding the area under the main crop with reference to farm | ①②③④⑤ |
| 142 | Investment in extending the farm size two-fold | ①②③④⑤ |
| 143 | Investment in buying more land for your farm | ①②③④⑤ |
| 144 | Investment in farm size de-fragmentation | ①②③④⑤ |
| 145 | Investment in the installation of your tube well | ①②③④⑤ |
| 146 | Investment in constructing a small water reservoir on the farm | ①②③④⑤ |
| 147 | Investment in livestock. | ①②③④⑤ |
| 148 | Investment in starting any alternative source of earning | ①②③④⑤ |
| 149 | Investment in increasing the labour force on the farm | ①②③④⑤ |
| 150 | Investment in learning new methods farming | ①②③④⑤ |
|  | **Rate the factors controlling the decision of your farming** |  |
| 151 | Changes in temperature | ①②③④⑤ |
| 152 | Changes in rainfall | ①②③④⑤ |
| 153 | Water availability | ①②③④⑤ |
| 154 | Amount of money/capital | ①②③④⑤ |
| 155 | Pest/insect attack | ①②③④⑤ |
| 156 | The market price of your crop | ①②③④⑤ |
| 157 | Government decisions and policies about farmers | ①②③④⑤ |
| 158 | Peers’ advice | ①②③④⑤ |
| 159 | Advice from agricultural extension services | ①②③④⑤ |
