# Supplementary material for: Sustainable livelihood capital and climate change adaptation in Pakistan's agriculture: Structural equation modeling analysis in the VIABLE framework
Source: Heliyon. 2023 Oct 13;9(11):e20818. doi: 10.1016/j.heliyon.2023.e20818 (PMC10623177; doi:10.1016/j.heliyon.2023.e20818)
Supplement: Multimedia component 2 [file mmc2.docx]

S2 Measurement Model

|  | **Cronbach's alpha** | **Composite reliability (rho_a)** | **Composite reliability (rho_c)** | **AVE** |
| --- | --- | --- | --- | --- |
| CFA | 0.742 | 0.796 | 0.837 | 0.569 |
| CM | 0.833 | 0.853 | 0.882 | 0.603 |
| EM | 0.673 | 0.686 | 0.802 | 0.505 |
| FC | 0.754 | 0.862 | 0.834 | 0.566 |
| FM | 0.725 | 0.77 | 0.844 | 0.645 |
| HC | 0.791 | 1.008 | 0.843 | 0.577 |
| HCO | 0.727 | 0.807 | 0.845 | 0.651 |
| IM | 0.644 | 0.662 | 0.809 | 0.587 |
| INC | 0.882 | 0.882 | 0.944 | 0.894 |
| INL | 0.821 | 0.85 | 0.917 | 0.847 |
| NC | 0.669 | 0.68 | 0.821 | 0.606 |
| NCO | 0.57 | 0.573 | 0.823 | 0.699 |
| NFA | 0.69 | 0.719 | 0.829 | 0.622 |
| SC | 0.826 | 0.854 | 0.873 | 0.537 |

**Outer Loadings**

|  | **Outer loadings** |
| --- | --- |
| CM100 <- CM | 0.658 |
| CM101 <- CM | 0.877 |
| CM102 <- CM | 0.84 |
| CM103 <- CM | 0.676 |
| CM105 <- CM | 0.808 |
| CO130 <- HCO | 0.6 |
| CO131 <- HCO | 0.907 |
| CO133 <- HCO | 0.878 |
| CO135 <- NCO | 0.851 |
| CO136 <- NCO | 0.821 |
| EM118 <- EM | 0.619 |
| EM120 <- EM | 0.783 |
| EM121 <- EM | 0.686 |
| EM123 <- EM | 0.743 |
| FA151 <- CFA | 0.911 |
| FA152 <- CFA | 0.823 |
| FA153 <- CFA | 0.587 |
| FA154 <- NFA | 0.833 |
| FA155 <- CFA | 0.651 |
| FA156 <- NFA | 0.87 |
| FA158 <- NFA | 0.645 |
| FC77 <- FC | 0.55 |
| FC78 <- FC | 0.644 |
| FC79 <- FC | 0.912 |
| FC80 <- FC | 0.846 |
| FM107 <- FM | 0.819 |
| FM108 <- FM | 0.883 |
| FM110 <- FM | 0.697 |
| HC81 <- HC | 0.639 |
| HC82 <- HC | 0.788 |
| HC83 <- HC | 0.725 |
| HC84 <- HC | 0.867 |
| IM112 <- IM | 0.717 |
| IM115 <- IM | 0.716 |
| IM116 <- IM | 0.857 |
| IN140 <- INC | 0.945 |
| IN141 <- INC | 0.946 |
| IN142 <- INL | 0.902 |
| IN143 <- INL | 0.938 |
| IN146 <- INW | 1 |
| NC89 <- NC | 0.682 |
| NC90 <- NC | 0.858 |
| NC92 <- NC | 0.785 |
| PUR45 <- V1 | 1 |
| PUR46 <- V2 | 1 |
| PUR47 <- V3 | 1 |
| PUR48 <- V4 | 1 |
| SC94 <- SC | 0.69 |
| SC95 <- SC | 0.834 |
| SC96 <- SC | 0.552 |
| SC97 <- SC | 0.751 |
| SC98 <- SC | 0.771 |
| SC99 <- SC | 0.768 |
