# Supplementary material for: Sustainable livelihood capital and climate change adaptation in Pakistan's agriculture: Structural equation modeling analysis in the VIABLE framework
Source: Heliyon. 2023 Oct 13;9(11):e20818. doi: 10.1016/j.heliyon.2023.e20818 (PMC10623177; doi:10.1016/j.heliyon.2023.e20818)
Supplement: Multimedia component 3 [file mmc3.docx]

**S3_ Discriminant validity**

**R-square**

|  | **R-square** | **R-square adjusted** |
| --- | --- | --- |
| CFA | 0.381 | 0.377 |
| CM | 0.6 | 0.593 |
| EM | 0.393 | 0.383 |
| FM | 0.493 | 0.485 |
| HCO | 0.219 | 0.215 |
| IM | 0.563 | 0.555 |
| INC | 0.534 | 0.531 |
| INL | 0.363 | 0.359 |
| INW | 0.407 | 0.403 |
| NCO | 0.124 | 0.119 |
| NFA | 0.549 | 0.545 |
|  |  |  |

**S3.1_Factor Loadings**

|  | CFA | CM | EM | FC | FM | HC | HCO | IM | INC | INL | INW | NC | NCO | NFA | SC | V1 | V2 | V3 | V4 |
| --- | --- | --- | --- | --- | --- | --- | --- | --- | --- | --- | --- | --- | --- | --- | --- | --- | --- | --- | --- |
| CM100 |  | 0.658 |  |  |  |  |  |  |  |  |  |  |  |  |  |  |  |  |  |
| CM101 |  | 0.877 |  |  |  |  |  |  |  |  |  |  |  |  |  |  |  |  |  |
| CM102 |  | 0.840 |  |  |  |  |  |  |  |  |  |  |  |  |  |  |  |  |  |
| CM103 |  | 0.676 |  |  |  |  |  |  |  |  |  |  |  |  |  |  |  |  |  |
| CM105 |  | 0.808 |  |  |  |  |  |  |  |  |  |  |  |  |  |  |  |  |  |
| CO130 |  |  |  |  |  |  | 0.600 |  |  |  |  |  |  |  |  |  |  |  |  |
| CO131 |  |  |  |  |  |  | 0.907 |  |  |  |  |  |  |  |  |  |  |  |  |
| CO133 |  |  |  |  |  |  | 0.878 |  |  |  |  |  |  |  |  |  |  |  |  |
| CO135 |  |  |  |  |  |  |  |  |  |  |  |  | 0.851 |  |  |  |  |  |  |
| CO136 |  |  |  |  |  |  |  |  |  |  |  |  | 0.821 |  |  |  |  |  |  |
| EM118 |  |  | 0.619 |  |  |  |  |  |  |  |  |  |  |  |  |  |  |  |  |
| EM120 |  |  | 0.783 |  |  |  |  |  |  |  |  |  |  |  |  |  |  |  |  |
| EM121 |  |  | 0.686 |  |  |  |  |  |  |  |  |  |  |  |  |  |  |  |  |
| EM123 |  |  | 0.743 |  |  |  |  |  |  |  |  |  |  |  |  |  |  |  |  |
| FA151 | 0.911 |  |  |  |  |  |  |  |  |  |  |  |  |  |  |  |  |  |  |
| FA152 | 0.823 |  |  |  |  |  |  |  |  |  |  |  |  |  |  |  |  |  |  |
| FA153 | 0.587 |  |  |  |  |  |  |  |  |  |  |  |  |  |  |  |  |  |  |
| FA154 |  |  |  |  |  |  |  |  |  |  |  |  |  | 0.833 |  |  |  |  |  |
| FA155 | 0.651 |  |  |  |  |  |  |  |  |  |  |  |  |  |  |  |  |  |  |
| FA156 |  |  |  |  |  |  |  |  |  |  |  |  |  | 0.870 |  |  |  |  |  |
| FA158 |  |  |  |  |  |  |  |  |  |  |  |  |  | 0.645 |  |  |  |  |  |
| FC77 |  |  |  | 0.550 |  |  |  |  |  |  |  |  |  |  |  |  |  |  |  |
| FC78 |  |  |  | 0.644 |  |  |  |  |  |  |  |  |  |  |  |  |  |  |  |
| FC79 |  |  |  | 0.912 |  |  |  |  |  |  |  |  |  |  |  |  |  |  |  |
| FC80 |  |  |  | 0.846 |  |  |  |  |  |  |  |  |  |  |  |  |  |  |  |
| FM107 |  |  |  |  | 0.819 |  |  |  |  |  |  |  |  |  |  |  |  |  |  |
| FM108 |  |  |  |  | 0.883 |  |  |  |  |  |  |  |  |  |  |  |  |  |  |
| FM110 |  |  |  |  | 0.697 |  |  |  |  |  |  |  |  |  |  |  |  |  |  |
| HC81 |  |  |  |  |  | 0.639 |  |  |  |  |  |  |  |  |  |  |  |  |  |
| HC82 |  |  |  |  |  | 0.788 |  |  |  |  |  |  |  |  |  |  |  |  |  |
| HC83 |  |  |  |  |  | 0.725 |  |  |  |  |  |  |  |  |  |  |  |  |  |
| HC84 |  |  |  |  |  | 0.867 |  |  |  |  |  |  |  |  |  |  |  |  |  |
| IM112 |  |  |  |  |  |  |  | 0.717 |  |  |  |  |  |  |  |  |  |  |  |
| IM115 |  |  |  |  |  |  |  | 0.716 |  |  |  |  |  |  |  |  |  |  |  |
| IM116 |  |  |  |  |  |  |  | 0.857 |  |  |  |  |  |  |  |  |  |  |  |
| IN140 |  |  |  |  |  |  |  |  | 0.945 |  |  |  |  |  |  |  |  |  |  |
| IN141 |  |  |  |  |  |  |  |  | 0.946 |  |  |  |  |  |  |  |  |  |  |
| IN142 |  |  |  |  |  |  |  |  |  | 0.902 |  |  |  |  |  |  |  |  |  |
| IN143 |  |  |  |  |  |  |  |  |  | 0.938 |  |  |  |  |  |  |  |  |  |
| IN146 |  |  |  |  |  |  |  |  |  |  | 1.000 |  |  |  |  |  |  |  |  |
| NC89 |  |  |  |  |  |  |  |  |  |  |  | 0.682 |  |  |  |  |  |  |  |
| NC90 |  |  |  |  |  |  |  |  |  |  |  | 0.858 |  |  |  |  |  |  |  |
| NC92 |  |  |  |  |  |  |  |  |  |  |  | 0.785 |  |  |  |  |  |  |  |
| PUR45 |  |  |  |  |  |  |  |  |  |  |  |  |  |  |  | 1.000 |  |  |  |
| PUR46 |  |  |  |  |  |  |  |  |  |  |  |  |  |  |  |  | 1.000 |  |  |
| PUR47 |  |  |  |  |  |  |  |  |  |  |  |  |  |  |  |  |  | 1.000 |  |
| PUR48 |  |  |  |  |  |  |  |  |  |  |  |  |  |  |  |  |  |  | 1.000 |
| SC94 |  |  |  |  |  |  |  |  |  |  |  |  |  |  | 0.690 |  |  |  |  |
| SC95 |  |  |  |  |  |  |  |  |  |  |  |  |  |  | 0.834 |  |  |  |  |
| SC96 |  |  |  |  |  |  |  |  |  |  |  |  |  |  | 0.552 |  |  |  |  |
| SC97 |  |  |  |  |  |  |  |  |  |  |  |  |  |  | 0.751 |  |  |  |  |
| SC98 |  |  |  |  |  |  |  |  |  |  |  |  |  |  | 0.771 |  |  |  |  |
| SC99 |  |  |  |  |  |  |  |  |  |  |  |  |  |  | 0.768 |  |  |  |  |
|  |  |  |  |  |  |  |  |  |  |  |  |  |  |  |  |  |  |  |  |

**S3.2_Fronell-Larcker Criterion (F&L)**

|  | CFA | CM | EM | FC | FM | HC | HCO | IM | INC | INL | INW | NC | NCO | NFA | SC | V1 | V2 | V3 | V4 |
| --- | --- | --- | --- | --- | --- | --- | --- | --- | --- | --- | --- | --- | --- | --- | --- | --- | --- | --- | --- |
| CFA | 0.754 |  |  |  |  |  |  |  |  |  |  |  |  |  |  |  |  |  |  |
| CM | 0.329 | 0.777 |  |  |  |  |  |  |  |  |  |  |  |  |  |  |  |  |  |
| EM | 0.279 | 0.396 | 0.711 |  |  |  |  |  |  |  |  |  |  |  |  |  |  |  |  |
| FC | 0.185 | 0.374 | 0.236 | 0.753 |  |  |  |  |  |  |  |  |  |  |  |  |  |  |  |
| FM | 0.329 | 0.600 | 0.509 | 0.146 | 0.803 |  |  |  |  |  |  |  |  |  |  |  |  |  |  |
| HC | 0.353 | 0.350 | 0.166 | 0.509 | 0.440 | 0.759 |  |  |  |  |  |  |  |  |  |  |  |  |  |
| HCO | -0.034 | -0.380 | -0.085 | -0.268 | -0.396 | -0.449 | 0.807 |  |  |  |  |  |  |  |  |  |  |  |  |
| IM | 0.223 | 0.530 | 0.441 | 0.527 | 0.398 | 0.161 | -0.202 | 0.766 |  |  |  |  |  |  |  |  |  |  |  |
| INC | 0.456 | 0.566 | 0.271 | 0.184 | 0.605 | 0.538 | -0.489 | 0.227 | 0.946 |  |  |  |  |  |  |  |  |  |  |
| INL | 0.342 | 0.107 | 0.499 | 0.388 | 0.133 | 0.109 | 0.055 | 0.395 | 0.106 | 0.920 |  |  |  |  |  |  |  |  |  |
| INW | 0.218 | 0.392 | 0.262 | 0.536 | 0.188 | 0.143 | -0.120 | 0.655 | 0.148 | 0.423 | 1.000 |  |  |  |  |  |  |  |  |
| NC | 0.398 | 0.568 | 0.477 | 0.516 | 0.452 | 0.440 | -0.307 | 0.467 | 0.541 | 0.471 | 0.360 | 0.778 |  |  |  |  |  |  |  |
| NCO | -0.155 | 0.003 | 0.104 | -0.130 | -0.216 | -0.333 | 0.520 | -0.015 | -0.156 | 0.021 | -0.037 | -0.045 | 0.836 |  |  |  |  |  |  |
| NFA | 0.439 | 0.407 | 0.264 | 0.122 | 0.488 | 0.513 | -0.298 | 0.131 | 0.705 | 0.087 | 0.013 | 0.463 | -0.079 | 0.789 |  |  |  |  |  |
| SC | 0.241 | 0.493 | 0.369 | 0.521 | 0.474 | 0.555 | -0.316 | 0.513 | 0.389 | 0.373 | 0.466 | 0.494 | -0.154 | 0.335 | 0.733 |  |  |  |  |
| V1 | 0.157 | 0.145 | 0.278 | 0.553 | -0.028 | 0.128 | 0.071 | 0.343 | -0.183 | 0.435 | 0.313 | 0.271 | 0.016 | -0.176 | 0.339 | 1.000 |  |  |  |
| V2 | 0.304 | 0.277 | 0.052 | 0.264 | 0.056 | 0.289 | -0.060 | 0.061 | 0.098 | -0.036 | 0.092 | 0.106 | 0.029 | 0.210 | 0.116 | 0.296 | 1.000 |  |  |
| V3 | 0.281 | 0.187 | 0.270 | 0.366 | -0.147 | -0.125 | 0.250 | 0.319 | -0.209 | 0.437 | 0.317 | 0.274 | 0.321 | -0.097 | 0.145 | 0.617 | 0.406 | 1.000 |  |
| V4 | 0.270 | 0.087 | -0.004 | 0.174 | 0.026 | 0.390 | 0.022 | -0.074 | 0.157 | 0.089 | -0.086 | 0.196 | -0.030 | 0.286 | 0.174 | 0.125 | 0.384 | 0.213 | 1.000 |

**S3.3_Cross Loadings**

|  | CFA | CM | EM | FC | FM | HC | HCO | IM | INC | INL | INW | NC | NCO | NFA | SC | V1 | V2 | V3 | V4 |  |
| --- | --- | --- | --- | --- | --- | --- | --- | --- | --- | --- | --- | --- | --- | --- | --- | --- | --- | --- | --- | --- |
| CM100 | 0.472 | 0.658 | 0.350 | 0.072 | 0.590 | 0.410 | -0.334 | 0.188 | 0.600 | 0.022 | 0.001 | 0.375 | -0.171 | 0.494 | 0.311 | -0.054 | 0.198 | -0.062 | 0.166 |  |
| CM101 | 0.142 | 0.877 | 0.283 | 0.383 | 0.437 | 0.190 | -0.321 | 0.533 | 0.397 | 0.053 | 0.458 | 0.432 | 0.064 | 0.231 | 0.381 | 0.157 | 0.262 | 0.194 | -0.047 |  |
| CM102 | 0.180 | 0.840 | 0.334 | 0.439 | 0.331 | 0.194 | -0.301 | 0.497 | 0.390 | 0.177 | 0.452 | 0.537 | 0.190 | 0.227 | 0.423 | 0.280 | 0.250 | 0.362 | 0.059 |  |
| CM103 | 0.345 | 0.676 | 0.338 | 0.093 | 0.620 | 0.405 | -0.338 | 0.209 | 0.565 | 0.014 | 0.032 | 0.419 | -0.157 | 0.503 | 0.380 | -0.095 | 0.119 | -0.105 | 0.093 |  |
| CM105 | 0.230 | 0.808 | 0.255 | 0.370 | 0.448 | 0.243 | -0.200 | 0.552 | 0.322 | 0.117 | 0.457 | 0.431 | -0.013 | 0.225 | 0.418 | 0.184 | 0.226 | 0.224 | 0.111 |  |
| CO130 | 0.138 | -0.286 | -0.086 | -0.602 | -0.107 | -0.186 | 0.600 | -0.517 | -0.092 | -0.225 | -0.454 | -0.376 | 0.290 | 0.026 | -0.317 | -0.291 | 0.045 | -0.125 | 0.183 |  |
| CO131 | -0.091 | -0.348 | -0.061 | -0.162 | -0.411 | -0.479 | 0.907 | -0.083 | -0.508 | 0.105 | 0.024 | -0.244 | 0.468 | -0.320 | -0.241 | 0.117 | -0.095 | 0.284 | -0.083 |  |
| CO133 | -0.056 | -0.295 | -0.072 | -0.067 | -0.366 | -0.363 | 0.878 | -0.058 | -0.474 | 0.138 | -0.030 | -0.196 | 0.474 | -0.326 | -0.254 | 0.200 | -0.054 | 0.316 | 0.035 |  |
| CO135 | -0.141 | 0.116 | 0.019 | -0.004 | -0.221 | -0.274 | 0.451 | 0.024 | -0.188 | -0.082 | 0.013 | -0.076 | 0.851 | -0.135 | -0.189 | 0.100 | 0.196 | 0.370 | 0.056 |  |
| CO136 | -0.117 | -0.120 | 0.162 | -0.223 | -0.137 | -0.284 | 0.419 | -0.052 | -0.067 | 0.126 | -0.079 | 0.003 | 0.821 | 0.009 | -0.064 | -0.081 | -0.162 | 0.158 | -0.113 |  |
| EM118 | 0.172 | 0.366 | 0.619 | 0.169 | 0.505 | 0.379 | -0.314 | 0.276 | 0.430 | 0.214 | 0.088 | 0.425 | -0.089 | 0.415 | 0.459 | 0.025 | -0.018 | -0.080 | 0.131 |  |
| EM120 | 0.180 | 0.167 | 0.783 | 0.233 | 0.208 | -0.027 | 0.047 | 0.378 | 0.047 | 0.528 | 0.223 | 0.374 | 0.166 | 0.054 | 0.152 | 0.282 | -0.044 | 0.352 | -0.061 |  |
| EM121 | 0.129 | 0.206 | 0.686 | 0.035 | 0.354 | 0.006 | 0.067 | 0.287 | 0.079 | 0.323 | 0.199 | 0.231 | 0.039 | 0.154 | 0.190 | 0.084 | -0.011 | 0.117 | -0.052 |  |
| EM123 | 0.294 | 0.394 | 0.743 | 0.194 | 0.420 | 0.136 | -0.054 | 0.303 | 0.234 | 0.316 | 0.224 | 0.313 | 0.140 | 0.167 | 0.273 | 0.338 | 0.207 | 0.306 | -0.018 |  |
| FA151 | 0.911 | 0.230 | 0.206 | 0.076 | 0.339 | 0.382 | -0.016 | 0.094 | 0.440 | 0.281 | 0.084 | 0.301 | -0.183 | 0.419 | 0.207 | 0.070 | 0.228 | 0.143 | 0.314 |  |
| FA152 | 0.823 | 0.262 | 0.143 | 0.081 | 0.290 | 0.291 | -0.072 | 0.107 | 0.418 | 0.101 | 0.213 | 0.212 | -0.206 | 0.325 | 0.108 | -0.041 | 0.252 | 0.030 | 0.167 |  |
| FA153 | 0.587 | 0.309 | 0.165 | 0.195 | 0.299 | 0.249 | -0.135 | 0.152 | 0.278 | 0.041 | 0.125 | 0.149 | -0.101 | 0.254 | 0.012 | 0.210 | 0.511 | 0.246 | 0.106 |  |
| FA154 | 0.393 | 0.386 | 0.291 | 0.017 | 0.457 | 0.389 | -0.199 | 0.075 | 0.621 | 0.121 | 0.018 | 0.357 | -0.013 | 0.833 | 0.275 | -0.146 | 0.243 | -0.036 | 0.231 |  |
| FA155 | 0.651 | 0.251 | 0.336 | 0.270 | 0.086 | 0.127 | 0.066 | 0.356 | 0.214 | 0.542 | 0.256 | 0.507 | 0.043 | 0.301 | 0.338 | 0.318 | 0.080 | 0.503 | 0.176 |  |
| FA156 | 0.376 | 0.300 | 0.265 | 0.056 | 0.434 | 0.422 | -0.260 | 0.080 | 0.585 | 0.044 | -0.024 | 0.369 | -0.086 | 0.870 | 0.227 | -0.191 | 0.161 | -0.165 | 0.201 |  |
| FA158 | 0.253 | 0.269 | 0.025 | 0.264 | 0.233 | 0.416 | -0.260 | 0.176 | 0.447 | 0.030 | 0.046 | 0.384 | -0.103 | 0.645 | 0.307 | -0.063 | 0.069 | -0.019 | 0.259 |  |
| FC77 | 0.284 | 0.358 | -0.023 | 0.550 | 0.238 | 0.646 | -0.353 | 0.161 | 0.450 | -0.056 | 0.063 | 0.349 | -0.221 | 0.448 | 0.396 | 0.081 | 0.255 | -0.023 | 0.384 |  |
| FC78 | -0.011 | 0.107 | 0.004 | 0.644 | 0.049 | 0.562 | -0.175 | 0.169 | 0.079 | 0.082 | 0.212 | 0.181 | -0.163 | 0.134 | 0.490 | 0.260 | 0.165 | 0.001 | 0.301 |  |
| FC79 | 0.166 | 0.393 | 0.332 | 0.912 | 0.178 | 0.358 | -0.192 | 0.552 | 0.125 | 0.479 | 0.569 | 0.511 | -0.029 | 0.034 | 0.497 | 0.633 | 0.231 | 0.473 | 0.057 |  |
| FC80 | 0.110 | 0.220 | 0.205 | 0.846 | -0.005 | 0.251 | -0.171 | 0.503 | 0.023 | 0.400 | 0.545 | 0.413 | -0.101 | -0.045 | 0.284 | 0.478 | 0.174 | 0.366 | 0.026 |  |
| FM107 | 0.180 | 0.403 | 0.356 | -0.002 | 0.819 | 0.304 | -0.309 | 0.222 | 0.494 | -0.011 | 0.072 | 0.257 | -0.202 | 0.420 | 0.285 | -0.223 | -0.003 | -0.300 | -0.028 |  |
| FM108 | 0.309 | 0.517 | 0.372 | 0.160 | 0.883 | 0.505 | -0.404 | 0.282 | 0.596 | 0.113 | 0.141 | 0.453 | -0.240 | 0.483 | 0.483 | -0.035 | 0.014 | -0.172 | 0.149 |  |
| FM110 | 0.313 | 0.551 | 0.555 | 0.205 | 0.697 | 0.192 | -0.209 | 0.520 | 0.324 | 0.252 | 0.277 | 0.371 | -0.040 | 0.231 | 0.358 | 0.250 | 0.155 | 0.191 | -0.119 |  |
| HC81 | 0.111 | 0.019 | -0.105 | 0.459 | 0.075 | 0.639 | -0.198 | 0.082 | 0.195 | 0.084 | 0.147 | 0.135 | -0.191 | 0.217 | 0.464 | 0.215 | 0.181 | -0.040 | 0.303 |  |
| HC82 | 0.293 | 0.185 | 0.062 | 0.407 | 0.220 | 0.788 | -0.235 | 0.011 | 0.302 | 0.070 | -0.018 | 0.279 | -0.231 | 0.330 | 0.357 | 0.149 | 0.284 | -0.047 | 0.362 |  |
| HC83 | 0.132 | 0.069 | 0.082 | 0.438 | 0.182 | 0.725 | -0.266 | 0.066 | 0.192 | 0.081 | 0.088 | 0.157 | -0.257 | 0.272 | 0.508 | 0.199 | 0.284 | -0.078 | 0.299 |  |
| HC84 | 0.383 | 0.481 | 0.253 | 0.371 | 0.557 | 0.867 | -0.496 | 0.220 | 0.647 | 0.096 | 0.175 | 0.521 | -0.304 | 0.552 | 0.444 | 0.007 | 0.195 | -0.149 | 0.290 |  |
| IM112 | 0.111 | 0.443 | 0.167 | 0.389 | 0.254 | 0.241 | -0.188 | 0.717 | 0.188 | 0.216 | 0.432 | 0.299 | -0.025 | 0.138 | 0.512 | 0.237 | 0.035 | 0.156 | 0.121 |  |
| IM115 | 0.261 | 0.408 | 0.643 | 0.292 | 0.472 | 0.072 | -0.089 | 0.716 | 0.195 | 0.454 | 0.475 | 0.418 | 0.023 | 0.100 | 0.352 | 0.274 | 0.000 | 0.282 | -0.143 |  |
| IM116 | 0.142 | 0.379 | 0.216 | 0.515 | 0.207 | 0.077 | -0.187 | 0.857 | 0.148 | 0.244 | 0.586 | 0.357 | -0.030 | 0.072 | 0.340 | 0.277 | 0.096 | 0.284 | -0.122 |  |
| IN140 | 0.435 | 0.565 | 0.243 | 0.181 | 0.566 | 0.497 | -0.455 | 0.204 | 0.945 | 0.023 | 0.118 | 0.505 | -0.106 | 0.659 | 0.329 | -0.154 | 0.113 | -0.175 | 0.157 |  |
| IN141 | 0.427 | 0.505 | 0.269 | 0.167 | 0.578 | 0.521 | -0.469 | 0.226 | 0.946 | 0.177 | 0.162 | 0.519 | -0.187 | 0.676 | 0.406 | -0.192 | 0.073 | -0.221 | 0.139 |  |
| IN142 | 0.296 | -0.026 | 0.391 | 0.380 | 0.003 | 0.049 | 0.087 | 0.351 | 0.025 | 0.902 | 0.373 | 0.369 | 0.033 | -0.001 | 0.270 | 0.457 | -0.015 | 0.441 | 0.063 |  |
| IN143 | 0.331 | 0.198 | 0.515 | 0.339 | 0.219 | 0.141 | 0.021 | 0.375 | 0.155 | 0.938 | 0.405 | 0.486 | 0.009 | 0.145 | 0.403 | 0.355 | -0.047 | 0.373 | 0.097 |  |
| IN146 | 0.218 | 0.392 | 0.262 | 0.536 | 0.188 | 0.143 | -0.120 | 0.655 | 0.148 | 0.423 | 1.000 | 0.360 | -0.037 | 0.013 | 0.466 | 0.313 | 0.092 | 0.317 | -0.086 |  |
| NC89 | 0.442 | 0.470 | 0.212 | 0.312 | 0.523 | 0.599 | -0.434 | 0.213 | 0.647 | 0.121 | 0.139 | 0.682 | -0.303 | 0.566 | 0.419 | 0.012 | 0.189 | -0.118 | 0.250 |  |
| NC90 | 0.195 | 0.521 | 0.469 | 0.484 | 0.319 | 0.254 | -0.227 | 0.472 | 0.363 | 0.416 | 0.391 | 0.858 | 0.091 | 0.277 | 0.368 | 0.285 | 0.098 | 0.320 | 0.075 |  |
| NC92 | 0.307 | 0.313 | 0.425 | 0.397 | 0.202 | 0.168 | -0.036 | 0.395 | 0.243 | 0.577 | 0.298 | 0.785 | 0.103 | 0.238 | 0.367 | 0.339 | -0.055 | 0.449 | 0.142 |  |
| PUR45 | 0.157 | 0.145 | 0.278 | 0.553 | -0.028 | 0.128 | 0.071 | 0.343 | -0.183 | 0.435 | 0.313 | 0.271 | 0.016 | -0.176 | 0.339 | 1.000 | 0.296 | 0.617 | 0.125 |  |
| PUR46 | 0.304 | 0.277 | 0.052 | 0.264 | 0.056 | 0.289 | -0.060 | 0.061 | 0.098 | -0.036 | 0.092 | 0.106 | 0.029 | 0.210 | 0.116 | 0.296 | 1.000 | 0.406 | 0.384 |  |
| PUR47 | 0.281 | 0.187 | 0.270 | 0.366 | -0.147 | -0.125 | 0.250 | 0.319 | -0.209 | 0.437 | 0.317 | 0.274 | 0.321 | -0.097 | 0.145 | 0.617 | 0.406 | 1.000 | 0.213 |  |
| PUR48 | 0.270 | 0.087 | -0.004 | 0.174 | 0.026 | 0.390 | 0.022 | -0.074 | 0.157 | 0.089 | -0.086 | 0.196 | -0.030 | 0.286 | 0.174 | 0.125 | 0.384 | 0.213 | 1.000 |  |
| SC94 | 0.023 | 0.257 | 0.161 | 0.505 | 0.077 | 0.382 | -0.143 | 0.343 | 0.088 | 0.191 | 0.334 | 0.239 | 0.058 | 0.077 | 0.690 | 0.458 | 0.212 | 0.258 | 0.120 |  |
| SC95 | 0.269 | 0.433 | 0.329 | 0.385 | 0.516 | 0.602 | -0.421 | 0.369 | 0.514 | 0.288 | 0.310 | 0.481 | -0.260 | 0.408 | 0.834 | 0.140 | 0.045 | -0.058 | 0.194 |  |
| SC96 | 0.364 | 0.261 | 0.084 | 0.180 | 0.312 | 0.477 | -0.198 | 0.175 | 0.363 | 0.050 | 0.092 | 0.245 | -0.210 | 0.443 | 0.552 | 0.100 | 0.185 | 0.033 | 0.342 |  |
| SC97 | 0.044 | 0.455 | 0.335 | 0.515 | 0.227 | 0.217 | -0.127 | 0.521 | 0.099 | 0.353 | 0.558 | 0.382 | 0.063 | 0.047 | 0.751 | 0.422 | 0.116 | 0.323 | 0.017 |  |
| SC98 | 0.120 | 0.394 | 0.357 | 0.454 | 0.261 | 0.213 | -0.053 | 0.510 | 0.123 | 0.438 | 0.489 | 0.409 | 0.074 | 0.082 | 0.771 | 0.384 | 0.007 | 0.330 | 0.072 |  |
| SC99 | 0.254 | 0.310 | 0.257 | 0.261 | 0.567 | 0.569 | -0.382 | 0.284 | 0.457 | 0.223 | 0.207 | 0.336 | -0.352 | 0.421 | 0.768 | 0.064 | 0.052 | -0.169 | 0.105 |  |
|  |  |  |  |  |  |  |  |  |  |  |  |  |  |  |  |  |  |  |  |  |

**S3.4_Heterotrait-monotrait Ratio of Correlations (HTMT)**

|  | CFA | CM | EM | FC | FM | HC | HCO | IM | INC | INL | INW | NC | NCO | NFA | SC | V1 | V2 | V3 | V4 |
| --- | --- | --- | --- | --- | --- | --- | --- | --- | --- | --- | --- | --- | --- | --- | --- | --- | --- | --- | --- |
| CFA |  |  |  |  |  |  |  |  |  |  |  |  |  |  |  |  |  |  |  |
| CM | 0.476 |  |  |  |  |  |  |  |  |  |  |  |  |  |  |  |  |  |  |
| EM | 0.390 | 0.544 |  |  |  |  |  |  |  |  |  |  |  |  |  |  |  |  |  |
| FC | 0.293 | 0.466 | 0.376 |  |  |  |  |  |  |  |  |  |  |  |  |  |  |  |  |
| FM | 0.503 | 0.818 | 0.781 | 0.258 |  |  |  |  |  |  |  |  |  |  |  |  |  |  |  |
| HC | 0.379 | 0.336 | 0.327 | 0.824 | 0.443 |  |  |  |  |  |  |  |  |  |  |  |  |  |  |
| HCO | 0.264 | 0.512 | 0.281 | 0.486 | 0.490 | 0.482 |  |  |  |  |  |  |  |  |  |  |  |  |  |
| IM | 0.361 | 0.708 | 0.673 | 0.710 | 0.633 | 0.311 | 0.397 |  |  |  |  |  |  |  |  |  |  |  |  |
| INC | 0.556 | 0.686 | 0.361 | 0.274 | 0.734 | 0.510 | 0.556 | 0.307 |  |  |  |  |  |  |  |  |  |  |  |
| INL | 0.411 | 0.179 | 0.644 | 0.431 | 0.250 | 0.128 | 0.259 | 0.546 | 0.148 |  |  |  |  |  |  |  |  |  |  |
| INW | 0.262 | 0.396 | 0.315 | 0.527 | 0.238 | 0.154 | 0.247 | 0.811 | 0.157 | 0.466 |  |  |  |  |  |  |  |  |  |
| NC | 0.585 | 0.749 | 0.702 | 0.670 | 0.639 | 0.492 | 0.507 | 0.708 | 0.701 | 0.637 | 0.435 |  |  |  |  |  |  |  |  |
| NCO | 0.328 | 0.321 | 0.322 | 0.312 | 0.311 | 0.467 | 0.793 | 0.091 | 0.215 | 0.184 | 0.072 | 0.346 |  |  |  |  |  |  |  |
| NFA | 0.601 | 0.569 | 0.443 | 0.376 | 0.648 | 0.605 | 0.463 | 0.233 | 0.899 | 0.110 | 0.045 | 0.695 | 0.188 |  |  |  |  |  |  |
| SC | 0.370 | 0.581 | 0.502 | 0.706 | 0.589 | 0.719 | 0.423 | 0.705 | 0.439 | 0.419 | 0.499 | 0.644 | 0.335 | 0.471 |  |  |  |  |  |
| V1 | 0.247 | 0.218 | 0.312 | 0.551 | 0.248 | 0.205 | 0.295 | 0.428 | 0.195 | 0.487 | 0.313 | 0.334 | 0.143 | 0.204 | 0.393 |  |  |  |  |
| V2 | 0.415 | 0.299 | 0.120 | 0.313 | 0.084 | 0.338 | 0.094 | 0.071 | 0.105 | 0.038 | 0.092 | 0.179 | 0.284 | 0.241 | 0.155 | 0.296 |  |  |  |
| V3 | 0.356 | 0.268 | 0.367 | 0.328 | 0.323 | 0.113 | 0.352 | 0.393 | 0.223 | 0.487 | 0.317 | 0.465 | 0.418 | 0.112 | 0.294 | 0.617 | 0.406 |  |  |
| V4 | 0.295 | 0.135 | 0.113 | 0.292 | 0.145 | 0.450 | 0.146 | 0.210 | 0.167 | 0.096 | 0.086 | 0.245 | 0.134 | 0.353 | 0.213 | 0.125 | 0.384 | 0.213 |  |

**S3.5_VIF**

|  | VIF |
| --- | --- |
| CFA | 1.000 |
| CM | 1.852 |
| Capital * CFA | 1.000 |
| Capital * NFA | 1.000 |
| EM | 1.495 |
| FC | 1.675 |
| FM | 1.811 |
| HC | 1.734 |
| HCO | 1.000 |
| IM | 1.522 |
| INL | 1.000 |
| INW | 1.000 |
| NC | 1.541 |
| NCO | 1.000 |
| NFA | 1.000 |
| PUR45 | 1.000 |
| PUR46 | 1.000 |
| PUR47 | 1.000 |
| PUR48 | 1.000 |
| SC | 1.759 |
