# Supplementary material for: Sustainable livelihood capital and climate change adaptation in Pakistan's agriculture: Structural equation modeling analysis in the VIABLE framework
Source: Heliyon. 2023 Oct 13;9(11):e20818. doi: 10.1016/j.heliyon.2023.e20818 (PMC10623177; doi:10.1016/j.heliyon.2023.e20818)
Supplement: Multimedia component 4 [file mmc4.docx]

S4_Structural Model (VIABLE-SEM)

|  | **Path coefficients** |
| --- | --- |
| CFA -> Adaptation | 0.168 |
| Capital -> Adaptation | 0.573 |
| Capital -> P1 | 0.596 |
| Capital -> P2 | 0.543 |
| HCO -> Adaptation | -0.257 |
| NCO -> Adaptation | 0.242 |
| NFA -> Adaptation | 0.036 |
| P1 -> V1 | 0.157 |
| P1 -> V2 | 0.131 |
| P1 -> V3 | 0.161 |
| P1 -> V4 | -0.151 |
| P2 -> V1 | 0.368 |
| P2 -> V2 | -0.091 |
| P2 -> V3 | 0.369 |
| P2 -> V4 | 0.152 |
| V1 -> Adaptation | 0.052 |
| V1 -> HCO | -0.133 |
| V1 -> NCO | -0.276 |
| V2 -> HCO | -0.187 |
| V2 -> NCO | -0.031 |
| V3 -> HCO | 0.387 |
| V3 -> NCO | 0.545 |
| V4 -> Adaptation | -0.142 |
| V4 -> HCO | 0.036 |
| V4 -> NCO | -0.075 |
| NFA x Capital -> Adaptation | -0.156 |
| CFA x Capital -> Adaptation | 0.05 |

|  | **Original sample (O)** | **Sample mean (M)** | **STDEV** | **T statistics** | **P values** | |
| --- | --- | --- | --- | --- | --- | --- |
| CFA -> Adaptation | 0.048 | 0.05 | 0.019 | 2.507 | | 0.006 |
| Capital -> Adaptation | 0.503 | 0.515 | 0.074 | 6.811 | | 0 |
| Capital -> P1 | 0.55 | 0.558 | 0.074 | 7.462 | | 0 |
| Capital -> P2 | 0.418 | 0.422 | 0.055 | 7.664 | | 0 |
| HCO -> Adaptation | 0.101 | 0.102 | 0.03 | 3.398 | | 0 |
| NCO -> Adaptation | 0.103 | 0.106 | 0.032 | 3.238 | | 0.001 |
| NFA -> Adaptation | 0.002 | 0.004 | 0.005 | 0.327 | | 0.372 |
| P1 -> V1 | 0.025 | 0.027 | 0.012 | 2.06 | | 0.02 |
| P1 -> V2 | 0.014 | 0.016 | 0.009 | 1.595 | | 0.055 |
| P1 -> V3 | 0.027 | 0.028 | 0.011 | 2.438 | | 0.007 |
| P1 -> V4 | 0.019 | 0.021 | 0.012 | 1.569 | | 0.058 |
| P2 -> V1 | 0.141 | 0.143 | 0.031 | 4.525 | | 0 |
| P2 -> V2 | 0.007 | 0.009 | 0.008 | 0.915 | | 0.18 |
| P2 -> V3 | 0.142 | 0.144 | 0.031 | 4.529 | | 0 |
| P2 -> V4 | 0.02 | 0.021 | 0.012 | 1.591 | | 0.056 |
| V1 -> Adaptation | 0.004 | 0.006 | 0.007 | 0.604 | | 0.273 |
| V1 -> HCO | 0.012 | 0.013 | 0.008 | 1.462 | | 0.072 |
| V1 -> NCO | 0.057 | 0.059 | 0.017 | 3.258 | | 0.001 |
| V2 -> HCO | 0.029 | 0.03 | 0.01 | 2.78 | | 0.003 |
| V2 -> NCO | 0.001 | 0.002 | 0.003 | 0.312 | | 0.377 |
| V3 -> HCO | 0.093 | 0.094 | 0.017 | 5.422 | | 0 |
| V3 -> NCO | 0.202 | 0.205 | 0.039 | 5.17 | | 0 |
| V4 -> Adaptation | 0.044 | 0.046 | 0.019 | 2.263 | | 0.012 |
| V4 -> HCO | 0.001 | 0.003 | 0.003 | 0.387 | | 0.349 |
| V4 -> NCO | 0.006 | 0.007 | 0.006 | 0.939 | | 0.174 |
| NFA x Capital -> Adaptation | 0.043 | 0.043 | 0.016 | 2.724 | | 0.003 |
| CFA x Capital -> Adaptation | 0.003 | 0.005 | 0.005 | 0.679 | | 0.248 |
