# Supplementary material for: Sustainable livelihood capital and climate change adaptation in Pakistan's agriculture: Structural equation modeling analysis in the VIABLE framework
Source: Heliyon. 2023 Oct 13;9(11):e20818. doi: 10.1016/j.heliyon.2023.e20818 (PMC10623177; doi:10.1016/j.heliyon.2023.e20818)
Supplement: Multimedia component 5 [file mmc6.docx]

**S6_**Collinearity_VIF_R2

**Table** Collinearity assessment, Variance Inflation Factor (VIF), and R squared.

| **Constructs** | **Variance Inflation Factor (VIF)** | **R squared (r^2^ )** |
| --- | --- | --- |
| Adaptation | .. | 0.62 |
| CM | 1.85 | .. |
| EM | 1.50 | .. |
| FM | 1.81 | .. |
| IM | 1.52 | .. |
| Capital | .. | .. |
| FC | 1.68 | .. |
| HC | 1.73 | .. |
| NC | 1.54 | .. |
| SC | 1.76 | .. |
| P1/INW | 1.00 | 0.35 |
| P2/INL | 1.00 | 0.29 |
| HCO | 1.00 | 0.10 |
| NCO | 1.00 | 0.17 |
| V1-PUR45 | 1.00 | 0.21 |
| V2-PUR46 | 1.00 | 0.02 |
| V3-PUR47 | 1.00 | 0.21 |
| V4-PUR48 | 1.00 | 0.03 |
| CFA | 1.00 | .. |
| NFA | 1.00 | .. |
| NFA x Capital | 1.00 | .. |
| CFA x Capital | 1.00 | .. |

The Variance Inflation Factor (VIF) measure of the PLSE-SEM indicates the multicollinearity issue. The higher multicollinearity indicates poor predictive accuracy of the model. It can lead to biased or unstable estimates of the regression coefficients, making it difficult to interpret the contribution of individual predictor variables to the model. The VIF value of less than ten indicates that multicollinearity is not a concern. Table 4 shows the VIF values of our model, which shows which is within the permissible limit.
